# From Clinical to Benchside: *Lacticaseibacillus* and *Faecalibacterium* Are Positively Associated With Muscle Health and Alleviate Age‐Related Muscle Disorder

**DOI:** 10.1111/acel.14485

**Published:** 2025-01-19

**Authors:** Chaoran Liu, Pui Yan Wong, Nilakshi Barua, Baoqi Li, Hei Yuet Wong, Ning Zhang, Simon Kwoon Ho Chow, Sunny Hei Wong, Jun Yu, Margaret Ip, Wing Hoi Cheung, Gustavo Duque, Christoph Brochhausen, Joseph Jao Yiu Sung, Ronald Man Yeung Wong

**Affiliations:** ^1^ Department of Orthopaedics & Traumatology The Chinese University of Hong Kong Hong Kong SAR China; ^2^ Department of Microbiology The Chinese University of Hong Kong Hong Kong SAR China; ^3^ Department of Orthopaedic Surgery Stanford University School of Medicine Palo Alto California USA; ^4^ Lee Kong Chian School of Medicine Nanyang Technological University Singapore Singapore; ^5^ Department of Medicine and Therapeutics The Chinese University of Hong Kong Hong Kong SAR China; ^6^ Bone, Muscle & Geroscience Research Group Research Institute of the McGill University Health Centre Montreal Quebec Canada; ^7^ Institute of Pathology, University Medical Center Mannheim, Heidelberg University Mannheim Germany

**Keywords:** aging, fecal microbiota transplantation, gut microbiota, muscle, probiotic, sarcopenia

## Abstract

Sarcopenia is an age‐related muscle disorder that increases risks of adverse clinical outcomes, but its treatments are still limited. Gut microbiota is potentially associated with sarcopenia, and its role is still unclear. To investigate the role of gut microbiota in sarcopenia, we first compared gut microbiota and metabolites composition in old participants with or without sarcopenia. Fecal microbiota transplantation (FMT) from human donors to antibiotic‐treated recipient mice was then performed. Specific probiotics and their mechanisms to treat aged mice were identified. Old people with sarcopenia had different microbial composition and metabolites, including *Paraprevotella*, *Lachnospira*, short‐chain fatty acids, and purine. After FMT, mice receiving microbes from people with sarcopenia displayed lower muscle mass and strength compared with those receiving microbes from non‐sarcopenic donors. *Lacticaseibacillus rhamnosus* (LR) and *Faecalibacterium prausnitzii* (FP) were positively related to muscle health of old people, and enhanced muscle mass and function of aged mice. Transcriptomics showed that genes related to tricarboxylic acid cycle (TCA) were enriched after treatments. Metabolic analysis showed increased substrates of TCA cycle in both LR and FP supernatants. Muscle mitochondria density, ATP content, NAD^+^/NADH, mitochondrial dynamics and biogenesis proteins, as well as colon tight junction proteins of aged mice were improved by both probiotics. LR and the combination of two probiotics also benefit intestinal immune health by reducing CD8^+^ IFNγ^+^ T cells. Gut microbiota dysbiosis is a pathogenesis of sarcopenia, and muscle‐related probiotics could alleviate age‐related muscle disorders mainly through mitochondria improvement. Further clinical translation is warranted.

## Graphical abstract

Old people with or without sarcopenia exhibited different gut microbiota and metabolite compositions. Mice receiving microbes from people with sarcopenia displayed lower muscle mass and strength compared with those receiving microbes from non‐sarcopenic donors. *Lacticaseibacillus rhamnosus* and *Faecalibacterium prausnitzii* were positively associated with muscle health in old people, and enhanced muscle mass and function of aged mice primarily by regulating mitochondria function and colon health.

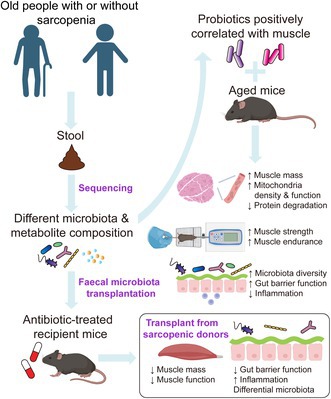

**Abbreviations**
ASMI: appendicular skeletal muscle mass index
AUC: area under curve
AWGS: Asian Working Group for Sarcopenia
BIA: bioelectrical impedance analysis
BSS: Bristol Stool Scale
CCI: Charlson Comorbidity Index
CFU: colony forming unit
CSA: cross‐sectional area
DXA: dual energy x‐ray absorptiometry
EDL: extensor digitorum longus
FMT: fecal microbiota transplantation
FP:
*Faecalibacterium prausnitzii*

GC–MS: gas chromatography–mass spectrometry
GM: gastrocnemius
H&E: Hematoxylin and Eosin
IBD: inflammatory bowel disease
LC–MS: liquid chromatography‐mass spectrometry
LDA: linear discriminant analysis
LEfSe: LDA effect size
LPS: lipopolysaccharide
LR: *Lacticaseibacillus rhamnosus*
MHC: myosin heavy chain
MSEA: metabolite set enrichment analysis
NMJ: neuromuscular junction
OPLS‐DA: orthogonal partial least squares discriminant analysis
OXPHOS: oxidative phosphorylation
PASE: Physical Activity Scale for the Elderly
QC: quality control
QUA: quadriceps
ROC: receiver operating characteristic
SCFAs: short‐chain fatty acids
SOL: soleus
TA: tibialis anterior
TCA: tricarboxylic acid cycle
TEM: transmission electron microscope
VIP: variables important in projection

## 1 Introduction

Sarcopenia is characterized by age‐related loss of muscle mass, strength, and poor physical performance. The prevalence of sarcopenia ranges from 10% to 27% in older persons, and is associated with adverse clinical outcomes, including fall, fracture, disability, and mortality (Chen et al. 2020; Petermann‐Rocha et al. 2021). Although resistance exercise and nutrition can attenuate sarcopenia, compliance of old individuals is largely problematic (Cruz‐Jentoft and Sayer 2019). Current research into etiology and treatment of sarcopenia are still limited. With the development of microbiota analysis techniques, including shotgun metagenomic analysis, 16S rDNA sequencing, and metabolomics, more evidence has shown that gut microbiota can influence health of hosts through regulation of various organs (Hou et al. 2022). The gut‐muscle axis is postulated to be involved in the development of sarcopenia due to gut microbiota dysbiosis during aging. This imbalance leads to bacteria derived inflammatory compounds traveling into the circulation affecting muscle metabolisms and mitochondria function (Liu et al. 2021). Pre‐clinical studies with germ‐free mice without gut microbiota had a significant reduction of muscle mass and strength, which could be reversed by microbiota transplantation (Lahiri et al. 2019). Probiotics as live microorganisms can improve health of hosts, and have potential for muscle growth through secreting beneficial metabolites (Giron et al. 2022). For instance, bacterial metabolites short‐chain fatty acids (SCFAs) are regulators of muscle health through modulating protein synthesis (Liu et al. 2024). Clinical studies demonstrated that old people with or without sarcopenia had different gut microbial composition (Wang et al. 2022). However, the causality of gut microbiota and sarcopenia is still unclear. Fecal microbiota transplantation (FMT) can be utilized to study causal relationship between gut microbes and disease (Gheorghe et al. 2021). Although bacterial products improved muscle mass in animals, translation to old individuals has not always been effective (Giron et al. 2022). Therefore, treatments obtained from clinical results may have better potential to translate back for clinical application. Since sarcopenia is strongly associated with colon disease (Faye et al. 2022), the improvement of colon health may also delay age‐related muscle impairment. Bacterial supplements are usually easy to consume and adhere to for old people, such as probiotic capsules and yogurt. It is therefore important to develop novel bacterial treatments for age‐related muscle disorders. In this study, (i) the gut microbiota composition and microbial metabolites in old people with or without sarcopenia were compared, (ii) FMT from human donors to recipient mice was utilized to detect causality between sarcopenia and gut microbiota, and for further clinical translation (iii) the potential therapeutic bacteria to treat sarcopenia were identified by confirming efficacy in aged mice.

## 2 Results

### 2.1 Demographics for Participants

To detect gut microbiota and metabolites related to sarcopenia, 51 older people (age 70.2 ± 5.0 years, 74.5% females) were recruited. Participants were separated into sarcopenic and non‐sarcopenic groups (28:23) according to Asian Working Group for Sarcopenia (AWGS) 2019 consensus (Chen et al. 2020). Body weight, body mass index (BMI), muscle mass, strength, and performance were significantly lower in older individuals with sarcopenia (Table S1), and other parameters were comparable. For daily nutrient intake, dietary fiber was reduced in people with sarcopenia (Table S2). 38 hip fracture patients were recruited for serum analysis (age 82.3 ± 6.7 years, 79% females), and were divided into low and high appendicular skeletal muscle mass index (ASMI) groups (19:19). Other physiological indicators were comparable (Table S3).

### 2.2 Gut Microbiota and Metabolites Are Different in Old People With or Without Sarcopenia

**FIGURE 1:**
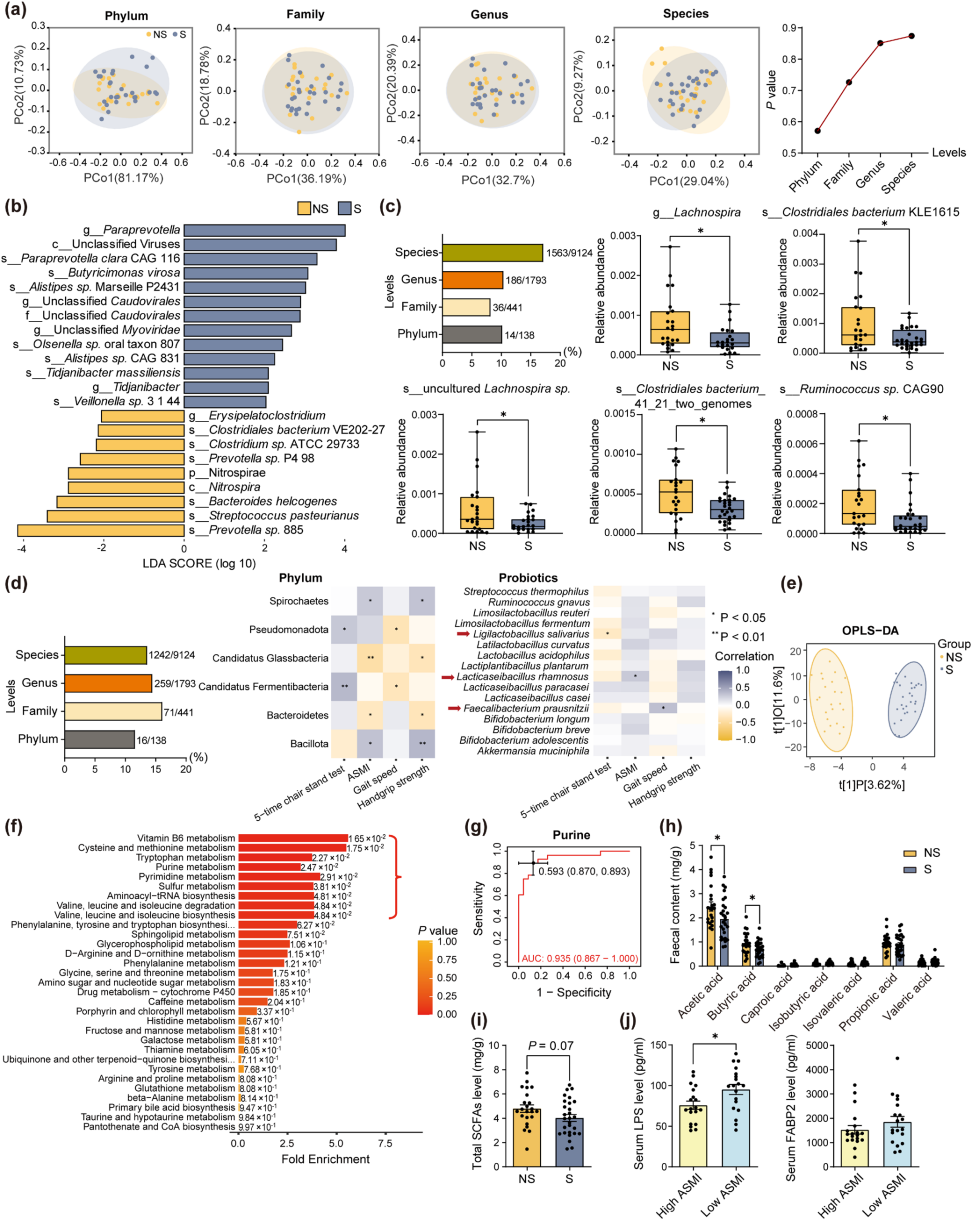
Characteristics of gut microbiota and metabolites in old people with sarcopenia. (a) Principal co‐ordinates analysis (PCoA) of gut microbiota at different levels between people with (S) or without sarcopenia (NS) based on Bray‐Curtis distances, and PERMANOVA tested with *p* value. (b) LEfSe analysis of relative abundance of gut microbiota between groups, LDA score (log 10) > 2, *p* < 0.05 by Wilcoxon test. (c) Percentage of differential gut microbiota between groups at different levels, and top abundant differential microbes. Significance is tested by Metastats analysis, *p < 0.05. (d) Percentage of gut microbiota that related to muscle indices at different levels, and phylum that related to at least 2 muscle indices, and the correlation between well‐known probiotics and muscle health. *p < 0.05, **p < 0.01, by Spearman correlation analysis. (e) OPLS‐DA plot derived from metabolomic sequencing of stools from people with or without sarcopenia. (f) Metabolite set enrichment analysis based on top 50 (low *p* value) differential metabolites between groups. Significance is set as *p* < 0.05. (g) ROC curves of purine to predict people with or without sarcopenia. AUC with 95% CI, and Youden index with specificity and sensitivity are shown. (h, i) Fecal SCFAs composition and total content between groups. (j) Serum LPS and FABP2 concentration as intestinal permeability biomarkers in hip fracture patients with lower or higher ASMI. Data are shown as means ± SEM (error bars). *p < 0.05, by two‐tailed, unpaired student's *t*‐test.

Shotgun metagenomic analysis was performed to compare diversity and relative abundance of gut microbiota between people with or without sarcopenia. Beta diversity showed no significant difference between groups (Figure 1a). Linear discriminant analysis (LDA) with effect size (LEfSe) showed that people with sarcopenia had higher abundance of genus *Paraprevotella*, *Butyricimonas virosa* , *Olsenella* sp. oral taxon 807, and lower relative abundance of species *Prevotella* sp. 885, *Clostridium* sp. ATCC 29733, *Clostridiales bacterium* VE202‐27 (Figure 1b). From Metastats analysis, 10.1% phylum, 8.2% family, 10.4% genus, and 17.1% species at each classification level were significantly different between groups (*q* < 0.05). Additionally, people with sarcopenia had lower abundance of genus *Lachnospira*, species *Clostridiales bacterium* KLE1615, uncultured *Lachnospira sp., Clostridiales bacterium*_41_21_two_ genomes, and *Ruminococcus* sp. CAG90 (*q* < 0.05; Figure 1c).

Further analysis was performed to investigate the association between gut microbiota abundance and muscle indicators that used to diagnose sarcopenia in old participants by Spearman correlation analysis. Over 10% taxonomy at each level were related to muscle health. Phylum Bacillota was positively related, whilst Bacteroidota was negatively related to muscle mass and handgrip strength. These correlations were also observed between two of these phyla and BMI (Figure S1a). In addition, family *Ruminococcaceae*, genus *Ruminococcus*, and its several species were also positively related to BMI, but species of *Bacteroides* were negatively related to BMI (Figure S1b–d). At species level, the focus was on the relationship between known probiotics and muscle health, allowing the selection of bacteria with minimal side effects. Amongst 16 probiotics, *Lacticaseibacillus rhamnosus* (LR) was positively associated with ASMI (*r* = 0.29, *p* = 0.038), and *Faecalibacterium prausnitzii* (FP) (*r* = 0.35, *p* = 0.013) and *Ligilactobacillus salivarius* (*r* = −0.29, *p* = 0.042) were related to better physical performance (Figure 1d).

Metagenomic analysis was performed on liquid chromatography‐mass spectrometry (LC–MS) to detect differential metabolites between people with or without sarcopenia. Orthogonal partial least squares discriminant analysis (OPLS‐DA) was utilized to explore the discrepancy of fecal metabolites between groups. Sarcopenic and non‐sarcopenic groups had different metabolite composition (Figure 1e). Variables important in projection (VIP), Log_2_ fold change (FC) score, and *p* value were combined to discover differential metabolites between groups. 32 gut microbial metabolites were significantly upregulated, and 5 downregulated in people with sarcopenia (Table S4). Microbial metabolites 5‐hydroxyhexanoic acid, ursodeoxycholic acid, tryptamine, and purine were increased in the sarcopenic group, whilst indole‐2‐carboxylic acid was reduced. Metabolite set enrichment analysis (MSEA) showed that vitamin B6, amino acids, purine metabolisms were different between two groups (Figure 1f). Receiver operating characteristic (ROC) curve showed that purine had the highest area under curve (AUC), with 0.935 (0.867–1.000) amongst the 37 metabolites (Log_2_FC = 1.27; *p* < 0.0001; Figure 1g).

SCFAs are beneficial gut microbiota metabolites. In participants with sarcopenia, acetic acid and butyric acid were significantly reduced, and a trend of lower total SCFAs was also found (Figure 1h,i). Serum concentrations of lipopolysaccharide (LPS) and fatty acid‐binding protein 2 (FABP2) can be biomarkers reflecting gut barrier function. People with history of a hip fracture have high prevalence of sarcopenia (Kim, Park, et al. 2022). A higher level of serum LPS but not FABP2 was found in hip fracture patients with low ASMI (Figure 1j).

### 2.3 Muscle Impairment Appeared in Recipient Mice After FMT From Human Donors With Sarcopenia

**FIGURE 2:**
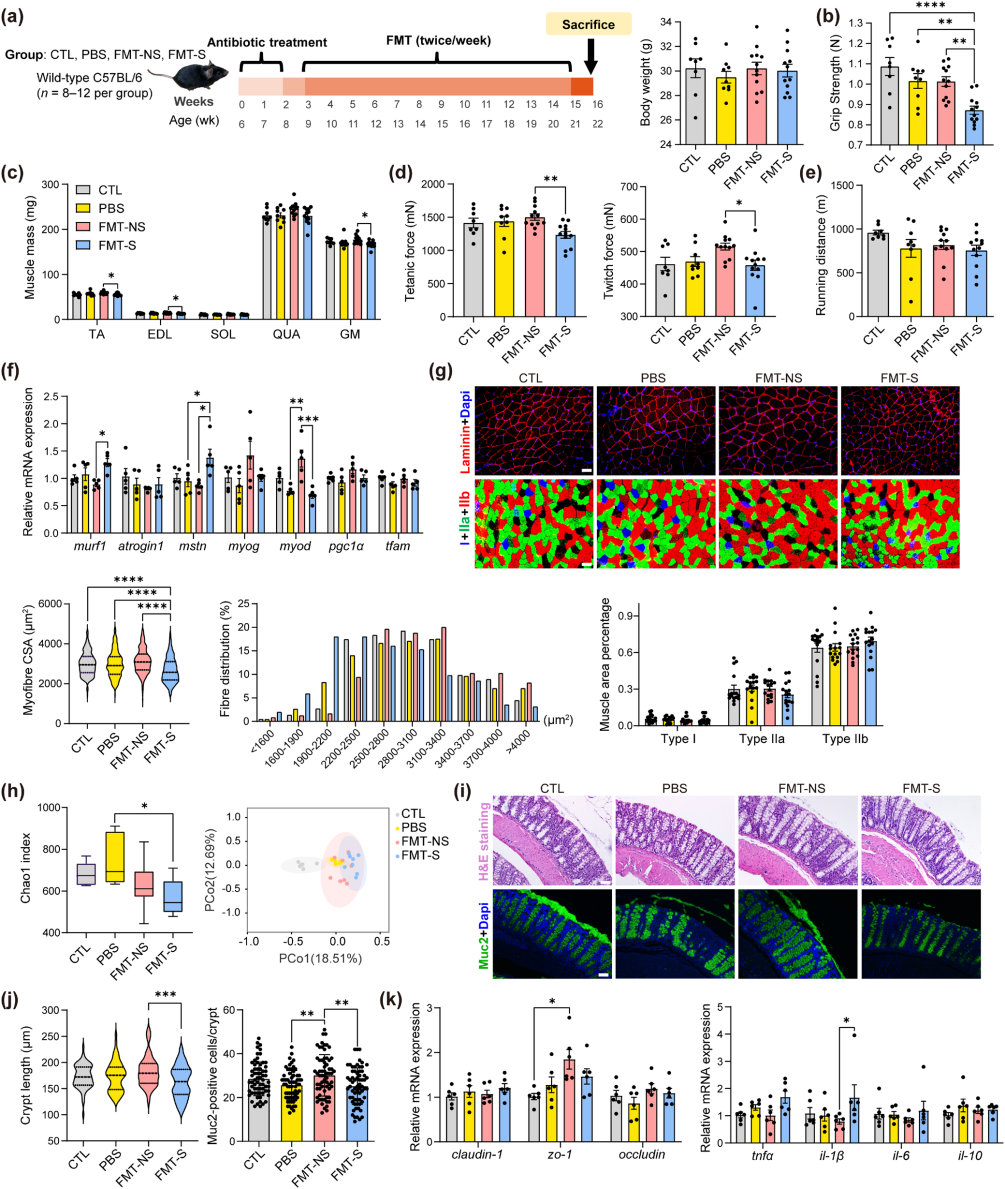
Muscle, colon, gut microbiota alterations of mice colonized microbiota from human donors with sarcopenia after FMT. (a) 6‐week‐old mice as control group (CTL group, *n* = 8), or received a two‐week antibiotic cocktail treatment in drinking water and 12‐week PBS (PBS group, *n* = 9) or solution gavage prepared by stool of human donors (FMT‐NS mice received from 4 non‐sarcopenic donors, and FMT‐S mice from 4 sarcopenic donors, 3 mice per donor, *n* = 12 mice per group) twice a week, and body weight between groups. (b) Grip strength of forelimbs. (c) Lower limb muscle wet weight at the endpoint (*n* = 8–12). (d) Twitch force and tetanic force tested of freshly isolated gastrocnemius (*n* = 8–12). (e) Treadmill running distance until exhaustion for anti‐fatigue capacity measurement (*n* = 8–12). (f) Expression of atrophic, myogenic, and mitochondria biogenesis genes at mRNA levels in randomly selected EDL muscles from each group (*n* = 5). (g) Immunofluorescence staining of gastrocnemius Laminin and MHC (Type I: Slow twitch; Type IIa: Intermediate twitch; Type IIb: Fast twitch). Images were taken at 20× magnification, scale bar = 50 μm. Muscle area percentage of different fiber types (*n* = 4 mice per group, myofibers and 16 positions of MHC staining per group were measured). Myofiber CSA and the fiber size distribution (*n* = 4 mice per group, 224–256 myofibers per group). Muscle area percentage of different fiber types (*n* = 4 mice, 16 positions of MHC staining per group). (h) Diversity and richness of the gut microbiota amongst groups including Chao1 index. Data are expressed by mean ± SEM. Significance is set as *p < 0.05, by Kruskal–Wallis with Dunn test. PCoA of gut microbiota amongst 4 groups based on Bray‐Curtis distances (*n* = 6–8 mice per group; for FMT groups, *n* = 2 mice per donor). (i, j) H&E and immunofluorescence staining of colon tissues (scale bar = 50 μm). Comparison of crypt length amongst groups (*n* = 5–8 mice per group, 70 crypts per group) and counts of Muc2‐positive cells per crypt amongst groups (*n* = 5–8 mice, 65 crypts per group). (k) mRNA expression of genes related to the gut barrier (*claudin‐1*, *zo‐1*, *occludin*) and cytokines (*tnfα*, *il‐1β*, *il‐6*, *il‐10*) in colon tissues (*n* = 6). Error bars indicate SEM. *p < 0.05, **p < 0.01, ***p < 0.001, ****p < 0.0001, by ANOVA followed by Bonferroni (a–e, g) or Tukey's (f, g, j, k) post hoc test.

Although gut microbiota composition was related to sarcopenia in the clinical study, the causal role remained unclear. In this study, muscle and gut status of antibiotic‐treated mice colonized with gut microbiota from people without sarcopenia (FMT‐NS group) and with sarcopenia (FMT‐S group) were compared. Control groups were mice receiving no treatment (CTL group) or with antibiotics and phosphate‐buffered saline (PBS) gavage (PBS group) (Figure 2a). At the baseline, mice from different groups had similar grip strength and body weight (Figure S2a), and 2‐week antibiotic treatment supressed weight gain and strength enhancement (Figure S2b). After treatments, FMT‐S mice had lower muscle wet weight, grip strength, and muscle force compared to FMT‐NS mice, but similar anti‐fatigue capacity (Figure 2b–e). Normalized muscle mass also decreased in the FMT‐S group (Figure S2c). FMT‐S mice had the smallest myofiber cross‐sectional area (CSA), higher expression of *murf1* and *mstn*, and lower *myod*, but similar fiber type percentage (Figure 2f,g).

The gut microbiota composition after transplantation was also detected by 16S rDNA sequencing. Alpha diversity indicated the richness of microbiota within a microbial community, and beta diversity measured the dissimilarity between two communities. Amongst groups, FMT‐S mice had lower Chao1 as alpha diversity than PBS mice, and beta diversity amongst groups was significantly different (*p* < 0.0001) (Figure 2h). The beta diversity was significant between FMT‐NS and FMT‐S groups when using Jaccard distance (*p* = 0.03). According to LEfSe, CTL group had the highest abundance of family Lachnospiraceae and genus *Clostridium*. FMT‐NS mice had higher family Akkermansiaceae, and genus *Faecalibacterium*, whilst FMT‐S mice had the highest phylum Bacteroidota. FMT‐S mice also had lower genus *Clostridium* than FMT‐NS mice (Figure S2d–f).

The gut microbiota in the intestinal lumen can affect colon health. It is known that gastrointestinal diseases contribute to muscle atrophy. FMT‐NS mice had longer colonic crypt length and more Muc2‐positive cells which can secrete mucus to protect gut barrier compared to FMT‐S (Figure 2i,j). Serum LPS concentration was comparable amongst all groups (Figure S2g), although higher *zo‐1* in FMT‐NS than CTL groups. *il‐1β* in the colon was lower in FMT‐NS compared to FMT‐S mice (Figure 2k).

### 2.4 Improvement of Muscle Mass, Function, and Myofiber After Probiotic Treatments

**FIGURE 3:**
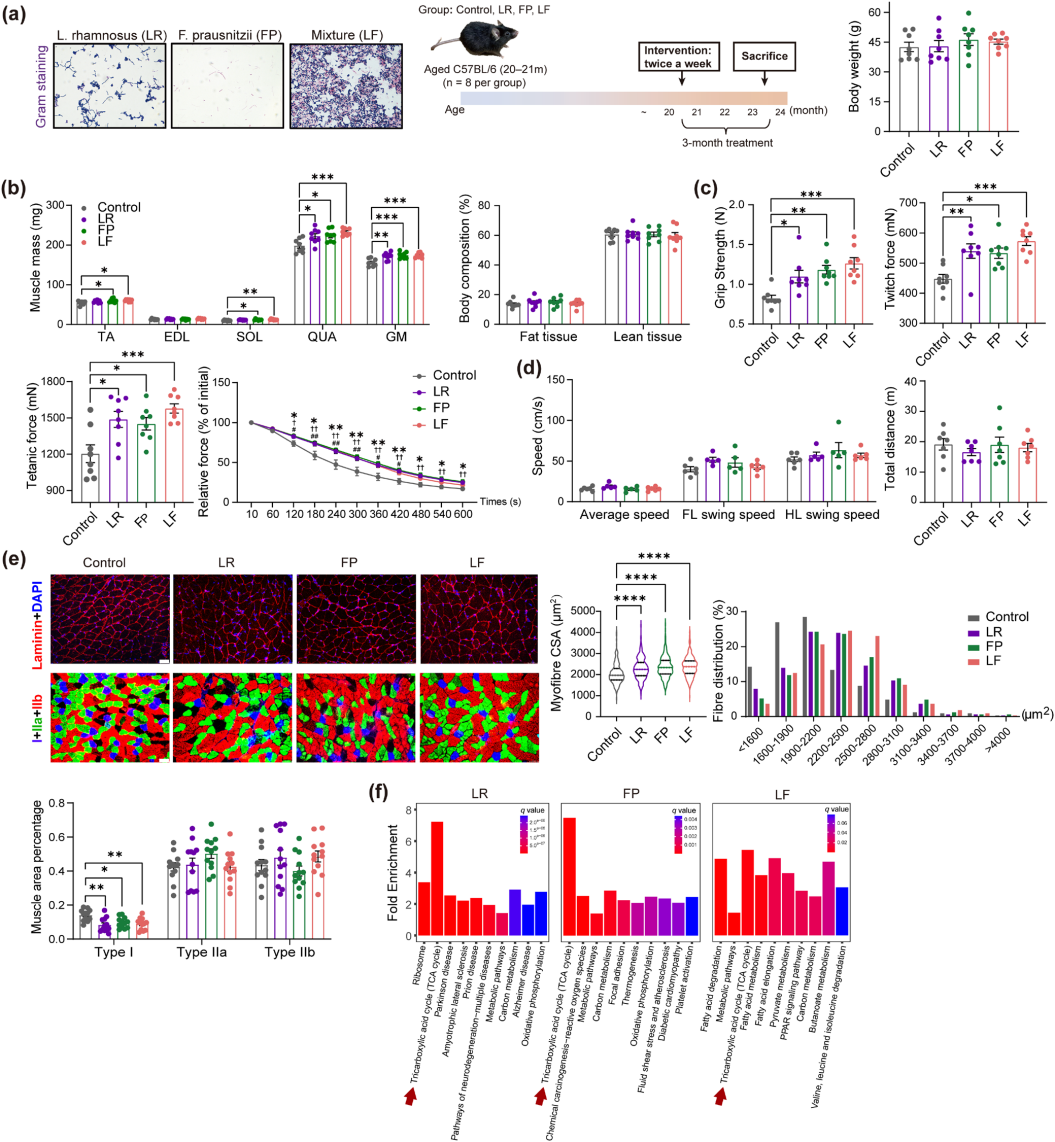
Muscle mass, strength, physical performance, myofiber structure, and muscle functional annotation of probiotic‐treated aged mice. (a) Gram staining of selected probiotics LR, FP and the combination (LF), and the administration of probiotics LR, FP, or LF to aged mice: 20–21 months old mice treated probiotics for 3 months and sacrificed at 23–24 months. Body weight at the endpoint (*n* = 8). (b) Lower limb muscle wet weight and body composition of total fat and lean tissue (*n* = 8). (c) Forelimb grip strength, gastrocnemius twitch and tetanic force, and anti‐fatigue capacity based on force descent rate (*n* = 8). *, ✝, # presented significant differences in LR, FP, or LF compared to the control groups. (d) Body gait speed, forelimb and hindlimb speed measured before sacrifice (*n* = 5–6). Total distance that mice traveled during 10 min in the open field (*n* = 7). (e) Immunofluorescence staining of gastrocnemius Laminin and MHC (scale bar = 50 μm). Myofiber CSA and the fiber size distribution (*n* = 3 mice per group, 330 myofibers per group). Muscle area percentage of different fiber types (*n* = 3 mice, 12 positions of MHC staining per group). All data expressed by mean ± SEM (error bar). *p < 0.05, **p < 0.01, ***p < 0.001, ****p < 0.0001 by ANOVA followed by Tukey's (a–e) or Bonferroni (d) post hoc test. (f) Top 10 KEGG functional annotation enrichment of three treatments (*n* = 4) according to differential genes between treated groups and the control group (*p* < 0.05). Red arrows point to TCA cycle. Significance is set as *p* < 0.05.

Gut microbiota from participants with sarcopenia did harm the muscle status of young recipient mice, but the colonization of microbes from people without sarcopenia did not bring more benefits to muscle health in recipient mice. Therefore, it is crucial to find specific probiotics that may be beneficial to muscle health to develop novel sarcopenia treatments. From clinical results, LR was the only probiotic positively correlated with muscle mass, and the positive correlation between FP abundance and muscle function was stronger than *Ligilactobacillus salivarius*. The abundance of LR and FP in human gut microbiota was not associated via Spearman analysis (*r* = −0.085, *p* = 0.555). To verify their potential as probiotics to attenuate age‐related muscle disorder, the interventional study was firstly conducted in mice. Aged mice (23–24 months old) had lower muscle mass, strength, gastrocnemius force, myofiber CSA, higher proportion of type I fiber, as well as poor colon health than young mice (5–6 months old), which could be used to study age‐related muscle disorders (Figure S3a–d). Age‐matched old mice were used, and the baseline (20–21 months) body weight and grip strength were similar amongst groups before being divided into different treated groups (Figure S4a). The body weight was comparable amongst groups (Figure 3a). After probiotic treatments, quadriceps (QUA) and gastrocnemius (GM) mass improved in all treated groups, whilst TA and soleus (SOL) mass only increased in FP and LF groups, but body composition and normalized muscle mass was comparable (Figure 3b, Figure S4b). Aged mice had increased grip strength, twitch and tetanic force after all treatments. According to the grip strength, treated groups did not had significant enhancement compared to the baseline, indicating probiotics could delay but not reverse sarcopenia (Figure S4c). The anti‐fatigue analysis showed that GM of treated groups had better anti‐fatigue capacity, but the resistance of LF group was comparable to the control group after 480s (Figure 3c). Gait speed and voluntarily traveled distance of mice were comparable (Figure 3d). Probiotic‐treated mice have larger muscle fiber CSA. Three probiotic‐treated groups reduced the area proportion of type I fiber compared to the control group (Figure 3e).

### 2.5 Increased Muscle Mitochondria Function Is Involved in Muscle Benefits From Probiotic Treatments

To identify mechanisms in how probiotics benefit muscle, transcriptome sequencing (RNA‐seq) was performed. Compared to the control group, genes with *p* < 0.05 in 3 treated groups were used to perform Kyoto Encyclopedia of Genes and Genomes (KEGG) functional annotations. From KEGG results, top functions of the LR group included tricarboxylic acid (TCA) cycle, metabolic pathways, and oxidative phosphorylation (OXPHOS). In the FP group, TCA cycle was most enriched. In the LF group, fatty acid degradation, metabolic pathways, TCA cycle were clustered (Figure 3f).

**FIGURE 4:**
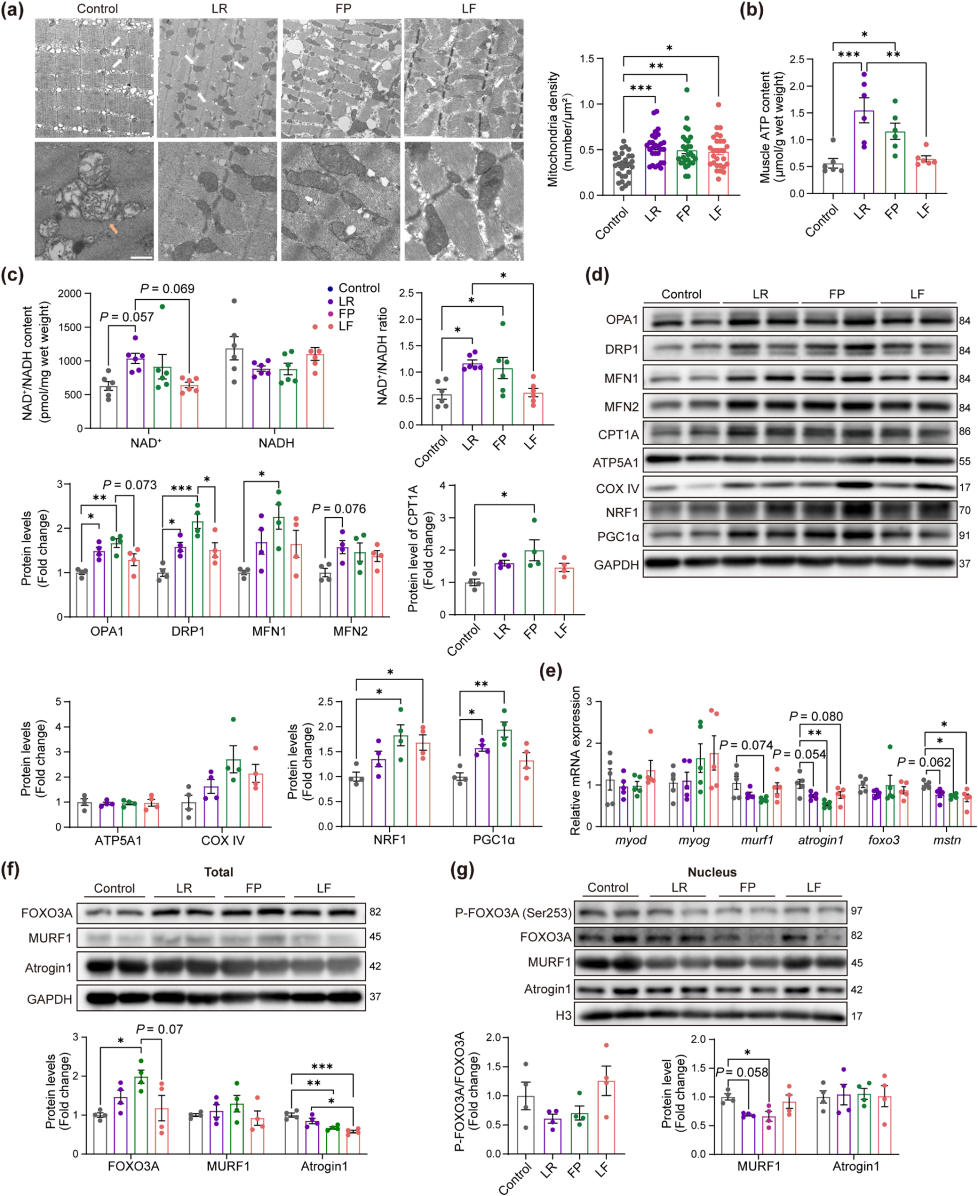
Mitochondria density, metabolism, and molecular changes in probiotic‐treated aged mice. (a) TEM micrographs of gastrocnemius muscle mitochondria (white arrow) amongst groups, and abnormal mitochondria structure in the control group (orange arrow) (scale bar = 500 nm). Mitochondria density (unit number) amongst groups (*n* = 4 mice per group, 28 images per group for analysis). (b) ATP content in quadriceps (*n* = 6). (c) Amount of NAD^+^ and NADH in quadriceps, and ratio of NAD^+^ to NADH amongst groups (*n* = 6). (d) Mitochondria‐related protein expression, fusion and fission: OPA1, DRP1, MFN1, MFN2; fatty acid oxidation: CPT1A; energy metabolism: ATP5A1, COX IV; biogenesis: NRF1, PGC1α in the total protein in TA muscles (*n* = 4). (e) Expression of myogenic, atrophic, and transcriptional regulator genes at mRNA levels in EDL muscles (*n* = 5 mice). (f) Atrophic and transcriptional regulator genes at total protein levels in TA muscles (*n* = 4). (g) Activation of FOXO3A and expression of atrophic genes at protein levels in nuclear of TA muscles (*n* = 4). All data are shown as means ± SEM (error bars). *p < 0.05, **p < 0.01, ***p < 0.001, by ANOVA followed by Tukey's post hoc test (a–g).

TCA cycle occurs in the mitochondrial matrix. Probiotics LR and FP had different metabolites compared to their culture medium (Figure S4d). They secreted several substances of TCA cycle, including oxaloacetate, succinic acid, pyruvic acid, cis‐aconitic acid, alpha‐ketoglutaric acid, L‐glutamine, 2‐oxoglutarate(2‐), and L‐malic acid (Figure S4e). In the animal study, the muscle mitochondria density of aged mice was lower than other groups, with more abnormal structures, including higher transparency, and reduced inner membrane folds (Figure 4a). ATP is generated by OXPHOS during TCA cycle and subsequent electron transport chain in the mitochondria. Aged mice treated with LR and FP alone had elevated muscle ATP content compared to the control group (Figure 4b). Aging is related to the reduction of NAD^+^/NADH ratio (Xie et al. 2020). Muscle NAD^+^/NADH ratio raised after LR and FP treatments (Figure 4c). For total muscle protein, the expression of mitochondrial fusion proteins OPA1 in LR and FP groups, MFN1 in the FP group, and fission protein DRP1 in LR and FP groups remarkedly increased. The fusion protein MFN2 was slightly improved in the LR group. The fatty acid oxidation‐related protein CPT1A was only increased in the FP group compared to control group. Other mitochondria metabolism‐related proteins, such as ATP5A1 and COX IV were similarly expressed between groups. Higher expression of mitochondria biogenesis proteins NRF1 in FP and LF groups was detected, as well as raised PGC1α in LR and FP groups (Figure 4d). The improvement of mitochondria function is closely related to muscle health enhancement after probiotic treatments.

### 2.6 Probiotics Reduce Molecules Related to Muscle Atrophy

Sarcopenia is associated with muscle atrophy, myogenesis impairment, and neuromuscular junction (NMJ) dysfunction. Related genes or proteins were detected. At mRNA levels, *gapdh* expression was not affected by probiotics (Figure S4f), and *atrogin1* significantly decreased in the FP group. LF and FP groups had lower *mstn*. Nevertheless, mRNA expression of *myod*, *myog*, and transcription factors *foxo3* was comparable (Figure 4e). At total protein levels, increased total FOXO3A was found by FP compared to control mice, and lower Atrogin1 expression was shown in FP and LF groups compared to control and LR groups (Figure 4f). Since E3 ubiquitin ligases was switched by FOXO3A in the nucleus, the nuclear protein was further detected. MURF1 was significantly decreased by FP, and a trend of lower MURF1 was found in the LR group. The activation level of FOXO3A and protein level of Atrogin1 were comparable in the nuclei (Figure 4g). NMJ function‐related genes showed no difference amongst groups (Figure S4g). FP showed resistance to muscle atrophy, and LF also slightly reduced atrophic markers. However, biomarkers of muscle hypertrophy and NMJ function were unaffected in all probiotic groups.

### 2.7 Probiotics Modulate Gut Microbiota Composition and Improve Gut Barrier, but Only LR and the Combination Enhance Intestinal Immunity

**FIGURE 5:**
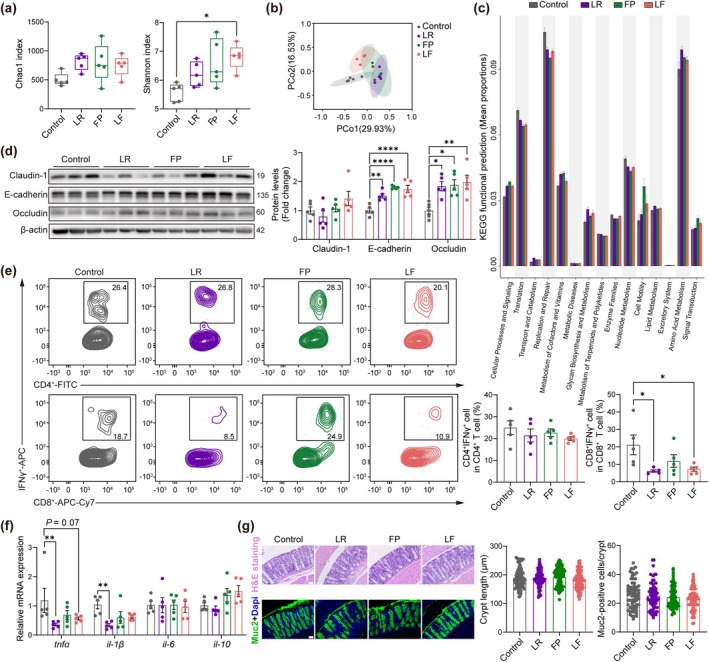
Gut microbial diversity and colon health of probiotic‐treated aged mice. (a) Diversity and richness of the gut microbiota amongst groups including Chao1 and Shannon indexes (*n* = 5). *p < 0.05, by Kruskal–Wallis followed by Dunn test. (b) PCoA of gut microbiota based on Bray‐Curtis distances (*n* = 5). (c) KEGG functional prediction via PICRUSt2. Functional prediction data with top 15 low *p* value, by Kruskal–Wallis test. (d) Expression of protein related to the gut barrier (*n* = 5). (e) Percentage of CD8^+^ IFNγ^+^ T cells in total CD8^+^ T cells, and CD4^+^ IFNγ^+^ T cells in total CD4^+^ T cells (*n* = 5). (f) mRNA expression of cytokines in colon tissues (*n* = 5). (g) H&E and Muc2‐positive cell staining of colon tissues, and the comparison of crypt length as well as counts of Muc2‐positive cells per crypt (*n* = 8 mice per group, 79 and 69 crypts per group, respectively) (Scale bar = 50 μm). All data are shown as mean ± SEM (error bar). *p < 0.05, **p < 0.01, ****p < 0.0001, by ANOVA followed by Tukey's post hoc test (d–g).

The gut microbiota and colon health might also be mediators of probiotic treatment and muscle status. Stools from mice were used to conduct gut microbiota 16S rDNA sequencing. Shannon index as alpha diversity showed richness of bacterial composition only increased in the LF group compared to control mice (Figure 5a). From beta diversity, three treated groups had different gut microbiota composition compared to the control group (*p* < 0.05; Figure 5b). Increased *Akkermansia* in FP and LR groups, and *Lachnospiraceae NK4A136 group* in LF and FP groups were found (*p* < 0.05) (Figure S5a). The functional annotation of the gut microbiota in the control group showed lower amino acid metabolism and lipid metabolism compared to the LR group, lower metabolism of vitamins compared to LR and FP groups, and higher metabolic diseases compared to FP and LF groups (*p* < 0.05) (Figure 5c).

The gut barrier‐related protein levels of E‐cadherin and Occludin increased after probiotic treatments, instead of Claudin‐1 (Figure 5d). A previous single‐cell study found that aged colon was related to IFNγ in CD4/8 T cells (Sirvinskas et al. 2022). To detect the colon immunity in aged mice, colon CD3^+^ cells were divided into CD4^+^ and CD8^+^ T cells (Figure S5b). A significant reduction of CD8^+^ IFNγ^+^ T cells was found in LR and LF groups, whilst CD4^+^ IFNγ^+^ T cells was comparable amongst groups (Figure 5e). Pro‐inflammatory cytokines *tnfα* and *il‐1β* in the colon reduced after LR treatment (Figure 5f). The histological structure of colon was similar amongst groups (Figure 5g). The rejuvenation of gut microbiome and increased levels of intestinal barrier function proteins were found in probiotic‐treated groups. LR effectively alleviated inflammation and immune disorders of the colon, and LF exhibited effects on microbial diversity and colon immune improvement.

## 3 Discussion

Sarcopenia is highly prevalent amongst older people (Cruz‐Jentoft, Bahat, et al. 2019). Evidence shows gut microbiota dysbiosis may contribute to the development of sarcopenia (Liu et al. 2021). In this study, dietary fiber intake that related to gut health was found to be significantly reduced in people with sarcopenia. People with sarcopenia had abundant genus *Paraprevotella*, which is related to sedentary behavior and low handgrip strength (Bressa et al. 2017; Zhao et al. 2023). Similar to a previous Korean study (Lee et al. 2022), increased *Butyricimonas* species was detected in the sarcopenic group. Species of *Lachnospira*, *Prevotella*, *Ruminococcus*, and *Clostridium* as butyrate producers were reduced in people with sarcopenia (Lee et al. 2022). In old people, Bacillota was positively associated with muscle health and BMI, whilst Bacteroidota was negatively associated. It was reported that old individuals with lower muscle mass had reduced ratio of Bacillota‐to‐Bacteroidota (Han et al. 2022).

The metabolomic analysis showed that old people with or without sarcopenia had potential different composition of gut microbial metabolites. People with obesity are less susceptible to sarcopenia (Liu et al. 2023). Secondary bile acid ursodeoxycholic acid as an anti‐adipogenesis molecule, was reasonable to enrich in people with lower BMI, such as sarcopenia (Oh et al. 2016). Indole‐2‐carboxylic acid, a metabolite positively related to genus *Faecalibaculum*, was enriched in people without sarcopenia (Zhang et al. 2022). Amongst the differential metabolites, purine was elevated in the sarcopenic group and could be a biomarker to distinguish older persons with or without sarcopenia. Lower acetic acid and butyric acid levels in people with sarcopenia were found, which is similar to previous findings using LC–MS (Guan et al. 2023). Exogenous supplements of SCFAs could attenuate sarcopenia in aged mice (Liu et al. 2024). By analyzing the serum LPS concentration, an increase in hip fracture patients with lower muscle mass was observed in this study. It represented that intestinal permeability may be impaired in people with muscle atrophy, and muscle dysfunction as reported before (Fielding et al. 2019).

FMT from older persons with or without sarcopenia to antibiotic‐treated mice to investigate causality between sarcopenia and gut microbiota was conducted. Stools from BMI‐ and age‐matched donors were selected for FMT, since not only age, but also BMI of donors could influence phenotypes of recipient mice (Fluitman et al. 2022). This antibiotic‐treated model has been used to reappear diseases through FMT, such as colorectal cancer (Wong et al. 2017). After antibiotic treatments, the richness and diversity of gut microbiota could significantly decrease (Ge et al. 2017). Similar to previous studies, antibiotics had a potential harmful effect on muscle (Bongers et al. 2022). Lower gain of weight and grip strength was found in mice after antibiotic treatments. Mice that received gut microbes from people with sarcopenia had impaired muscle phenotypes. Previous studies indicated that muscle strength of mice could be affected by FMT from older human donors with different physical function (Fielding et al. 2019). In this study, mice received microbiota from donors with sarcopenia had lower muscle mass, strength, myofiber CSA, mRNA expression of *myod* as well as higher *murf1* and *mtsn*. Based on gut microbiota analysis, PBS‐treated mice had higher alpha diversity compared to FMT‐S group, which may benefit from the self‐adjustment of gut microbiota. Nevertheless, FMT from sarcopenic donors impeded the recovery. Beta diversity showed the microbial composition between FMT‐S and FMT‐NS groups was different. Compared to control and FMT‐NS groups, antibiotic and gut microbiota from sarcopenic donors reduced *Akkermansia* and *Desulfovibrio*, elucidating the better trajectory induced by gut microbiota from non‐sarcopenia donors (Ge et al. 2017). Despite comparable circulatory LPS levels, FMT‐S mice had fewer Muc2‐positive cells than FMT‐NS mice indicating the gut barrier impairment. The higher expression of pro‐inflammatory cytokine *il‐1β* in FMT‐S mice might be induced by the impaired intestinal barrier and microbial dysbiosis. Therefore, both muscle and colon were negatively affected by the gut microbiota of sarcopenic donors. Similar to the finding from a Mendelian randomization study (Zhang et al. 2024), the reappearance of the sarcopenic phenotype in recipient mice following gut microbiota transplantation indicates that gut microbiota dysbiosis is a cause of sarcopenia.

Although it has been reported that microbes from young mice enhanced muscle function and fiber CSA of old mice (Kim, Chung, et al. 2022), gut microbiota from old people without sarcopenia did not improve muscle health significantly. This indicated that bacteria enriched in non‐sarcopenic donors as treatments may not be entirely useful. But it suggested that certain bacteria from sarcopenic donors may be responsible for sarcopenia. However, identifying novel treatments to reduce specific bacteria that enriched in sarcopenia is difficult. Previous studies have shown that multi‐probiotic supplements were safe to use and could improve muscle strength and alleviate intestinal leak in people with sarcopenia (Qaisar et al. 2024). Probiotics that can benefit both muscle mass and strength are warranted. In this study, LR and FP were selected as novel treatments since their abundance was both positively associated with muscle mass or function. It has been reported that LR alone could improve muscle strength and fiber CSA in young mice (Lee et al. 2023). The strain of LR that we chose has already been used clinically, which had high translational value as a treatment for sarcopenia (Wischmeyer et al. 2024). FP was the most reported bacterium that is positively related to muscle health in old people, and could enhance muscle mass and mitochondria function in obese mice (Lv et al. 2021; Munukka et al. 2017). The FP strain used in this study had beneficial effects on colon barrier function (Kawade et al. 2019). Similar to previous methods, the effect of bacteria correlated with human muscle indicators was verified in animal experiments (Blanton et al. 2016). Aged male mice were used since they were less affected by hormone fluctuation and had more obvious muscle disorders than female mice, such as mitochondria dysfunction (Kerr et al. 2024; Xie et al. 2021). Two selected probiotics and their combination had been found to be associated with improvement of muscle mass, strength, and myofiber size in old mice. The anti‐fatigue capacity of muscle also improved mainly in LR and FP groups according to the lower descent rate of muscle force after 10 min stimulations. There were no observable changes in the physical performance of mice after treatments. Age‐related alterations of muscle fiber types from II to I also reduced in treated groups. From functional annotation of muscle RNA‐seq, TCA cycle, OXPHOS, or fatty acid metabolism were regarded as potential mechanisms. Mitochondria are the primary site of these processes. Additionally, both probiotics secreted substances of TCA cycle, which may be utilized by administrated mice. Higher density and normal structure of mitochondria were found in all treated groups. ATP content and NAD^+^/NADH also improved after LR and FP treatments. Similar to RNA‐seq results, the mitochondria energy metabolism in the LF group was not as high as other treated groups, which may elucidate its lower anti‐fatigue capacity. Proteins related to mitochondria fusion and fission were highly expressed in LR‐ and FP‐treated mice, indicating the dynamic of mitochondria was higher. Since mitochondria dysfunction is one of the most important pathogeneses of sarcopenia (Kerr et al. 2024), the improvement of mitochondria function by probiotics indicated that gut microbiota regulation is an approach to delay sarcopenia. Probiotic‐treated groups also had higher total protein level of proteins related to mitochondria biogenesis. FOXO3A as an important transcription factor, was significantly reduced with age (Jing et al. 2023), and improved by FP. The muscle‐specific *mstn* at mRNA level and Atrogin1 at total protein level were reduced in FP and LF groups, and reduced MURF1 at nuclear protein level was only found in the FP groups. Therefore, decreased protein degradation was also an important mechanism of muscle growth by probiotic treatments. Probiotics used in this study did not affect NMJ function. All probiotics benefit muscle mass and strength of aged mice. In molecular levels, probiotic treatments attenuated muscle mitochondria dysfunction and slightly reduced atrophy, in which LR most significantly improved mitochondria metabolism, and FP exhibited the most significant reduction of muscle atrophy.

The gut microbiota and colon health that may be regulators of muscle were further analyzed. The disease of colon, such as inflammatory bowel disease (IBD) is associated with sarcopenia (Faye et al. 2022). LF promoted the alpha diversity, and all treatments altered the gut microbiota composition. LR‐ and FP‐treated mice had higher abundance of *Akkermansia*, which is important for colon health in aged mice (van der Lugt et al. 2019). *Lachnospiraceae NK4A136 group* as a potential butyrate producer also increased in FP and LF groups (Stadlbauer et al. 2020). The expression of gut barrier‐related proteins E‐cadherin and Occludin was increased, indicating improved gut barrier function. However, the number of Muc2‐positive cells did not change. Colon inflammation levels ameliorated in the LR group, which was also slightly reduced in the LF but not in the FP group. Elevated CD8^+^ IFNγ^+^ T cells as main age‐related changes of colon (Sirvinskas et al. 2022), were reduced in both LR and LF groups. Probiotic LR and LF significantly improved colon immune and reversed age‐related changes of colon. Prevention of colon diseases can remarkedly reduce the incidence of sarcopenia. Therefore, the improvement of colon health by probiotics is also an approach to promote muscle strength and mass.

There are several limitations of this study. The detection of circulating gut microbiota‐derived metabolites was not performed. Serum markers of gut barrier function were only detected in hip fracture patients with high or low muscle mass, which may have bias to interpret changes of sarcopenia. Further studies should compare these parameters in old people with or without sarcopenia. A large‐scale study is warranted to identify differential gut microbiota and metabolites with higher accuracy. Recipient mice used for FMT in this study were antibiotic‐treated mice. As antibiotics may have harmful effects on muscle at early time‐periods, germ‐free mice were recommended to use in the future, which can minimize confounding factors (Liu et al. 2021). For the therapeutic studies, only aged male mice were used, which may have different effect in female mice and humans. However, as treatment strategies in this study were developed based on clinical outcomes, there is great potential for selected probiotics to be clinically translated in patients with sarcopenia. Novel treatments that could reduce bacteria enriched in people with sarcopenia should be developed. Additionally, this study did not use sarcopenic mice with muscle indicators 2SD below the mean of young mice (Kerr et al. 2024). Further studies should use sarcopenic mice to mimic the status of sarcopenia in humans.

In conclusion, sarcopenia is an age‐related muscle disorder closely associated with the gut microbiota. The metabolite purine could accurately distinguish these two groups. FMT from people with sarcopenia impaired muscle mass, strength, as well as colon health in recipient mice, indicating gut microbiota dysbiosis as a pathogenic factor of sarcopenia. The probiotics LR and FP were identified to be positively related to human muscle mass and function, and these probiotics were used in aged mice which later showed improvement in muscle mass, strength, and reverse of fiber type changes. Enhanced muscle mitochondria function and slightly reduced atrophic levels were involved in all probiotic treatments. Although all treatments were beneficial to gut barrier function, only LR and LF are effective in improving colon immune. Differential gut microbiota and metabolites may be biomarkers for early sarcopenia detection in old people, and the regulation of gut microbiota may be a therapeutic strategy for sarcopenia. It is expected that probiotics LR and FP or their beneficial metabolites further investigated as sarcopenia treatments in future randomized controlled trials.

## 4 Materials and Methods

### 4.1 Participant Recruitment

Participants were recruited from the community or the Prince of Wales Hospital in Hong Kong from May 2021 to November 2021. Inclusion and exclusion criteria were shown in Supporting Information. The study was approved by The Joint Chinese University of Hong Kong—New Territories East Cluster Clinical Research Ethics Committee (CREC Ref No. 2021.008). Sarcopenia was diagnosed following recommendations of AWGS 2019 consensus (Chen et al. 2020). Details seen in Supporting Information.

Blood was collected from hip fracture patients (age > 60 years) that underwent an operation at the Prince of Wales Hospital from September 2021 to October 2022. After operation, participants were examined with dual energy x‐ray absorptiometry (DXA) for assessing muscle mass when vital signs were stable. ASMI was calculated and used to divide hip fracture participants into high (top 50%) or low ASMI (the rest 50%) groups.

### 4.2 Questionnaire Assessments

Physical Activity Scale for the Elderly (PASE), Charlson Comorbidity Index (CCI), Bristol Stool Scale (BSS), food frequency questionnaires (FFQ) and self‐reported questionnaire were recorded. Details seen in Supporting Information.

### 4.3 Shotgun Metagenomic Analysis

Stool samples from participants with or without sarcopenia were performed metagenomic analysis. Details seen in Supporting Information. Quality control (QC) of all sequencing data was shown in Supporting Information.

### 4.4 FMT Treatment of Antibiotic‐Treated Mice

Stool samples from 4 sarcopenic and 4 non‐sarcopenic BMI‐ (ranged 18.0–24.0 kg/m^2^) and gender‐matched human donors were diluted with sterile cold PBS at 0.2 g/mL to prepare gavage solution, respectively. 6‐week‐old male C57BL/6 mice were divided into 4 groups (*n* = 8–12 per group). The control (CTL) group received no treatment, whilst PBS and another 2 FMT groups were firstly treated with antibiotic cocktails. Details seen in Supporting Information. FMT of microbiota from non‐sarcopenic and sarcopenic human donors was conducted in antibiotic‐treated mice which were randomly divided into 8 cages (*n* = 3 per cage) as FMT‐NS and FMT‐S groups. After 2‐week antibiotic treatment and 1‐week washout period, mice were gavaged with 200 μL sterile PBS or suspension containing the gut microbiota twice a week for 12 weeks. The study was approved by the Animal Experimentation Ethics Committee of The Chinese University of Hong Kong (Ref: 20‐284‐MIS).

### 4.5 Probiotic Preparation and Treatment for Aged Mice

*Lacticaseibacillus rhamnosus* ATCC 53103 (LR) and *Faecalibacterium prausnitzii* ATCC 27768 were purchased from the American Type Culture Collection (ATCC) and cultured. Details seen in Supporting Information.

Muscle indicators of male aged C57BL/6 mice (23–24 months) were detected to verify the occurrence of aged‐related muscle disorder by comparing to young mice (5–6 months) (*n* = 6 per group). 20–21 months old male C57BL/6 mice were separated into 4 groups (*n* = 8 per group) for probiotic interventions: mice without receiving any treatment were in the control group, mice received 200 μL solution with 2 × 10^9^ CFU LR was in the LR group, 2 × 10^9^ CFU FP was in the FP group, and a mixture of 1 × 10^9^ CFU LR and 1 × 10^9^ CFU FP were in the LF group. Mice were orally gavaged with freshly prepared probiotics twice a week for 3 months. These two probiotics administrated for twice a week in mice were previously reported beneficial to muscle or intestine health, and 2 × 10^9^ CFU probiotic was safe for mice (Munukka et al. 2017; Si et al. 2022). For all animal studies, each batch of mice was raised together until divided into different treated groups to ensure similar gut microbiota composition at the baseline (Figure S6a,b). Baseline information of young and aged mice for FMT and probiotic studies were measured.

### 4.6 DNA Extraction and 16S rDNA Sequencing

Details seen in Supporting Information.

### 4.7 LC–MS and GC–MS Analyses

The LC–MS analysis was performed to detect the metabolites of human stool samples and probiotic supernatant. The content SCFAs was detected by GC–MS analysis. Details seen in Supporting Information.

### 4.8 ELISA Analysis of Serum

Blood samples from hip fracture patients as well as mice were centrifuged to separate serum. Human LPS and FABP2, and mouse LPS ELISA kits were used following the manual instruction to detect the concentration.

### 4.9 Lean Mass and Muscle Wet Weight

The whole‐body lean mass of mice was analyzed by animal DXA under anesthesia before sacrifice. Briefly, mice were placed in the center of the machine with limbs extended. The setting of high KV is 40, and high UA is 1000, whilst low KV is 20, and low UA is 1000. The percentage of fat tissue and lean tissue was obtained. After dissection, muscle from both legs were extracted for weighing, ex vivo functional test, tissue sectioning, or stored at −80°C for further analysis. Muscle wet weight of left TA, EDL, SOL, QUA, and GM were assessed as muscle mass. The body weight of mice was also recorded, and the normalized muscle mass was calculated.

### 4.10 Muscle Functional Tests

Forelimb grip strength, ex vivo muscle function, treadmill endurance, and open filed tests were conducted. Details seen in Supporting Information.

### 4.11 Immunofluorescence Staining

Gastrocnemius and colon sections were prepared for immunofluorescence staining, including muscle myosin heavy chain (MHC) staining and Laminin staining, and colon Muc2 staining. Hematoxylin and Eosin (H&E) staining was also conducted in colon tissues. Details seen in Supporting Information.

### 4.12 Transcriptome Sequencing

Details seen in Supporting Information.

### 4.13 Mitochondria Density Assessment

The mice gastrocnemius was prepared for mitochondria density assessment by transmission electron microscope (TEM). Details seen in Supporting Information.

### 4.14 ATP and NAD ^+^/NADH Detection

ATP Assay Kit and NAD/NADH Assay Kit were used according to the manufacturer's procedure to detect ATP, NAD^+^ and NADH content, and the ratio of NAD^+^/NADH in quadriceps.

### 4.15 Flow Cytometry of Colon T Cells

Fresh colon tissues were prepared for T cell detection. Anti‐CD3, anti‐CD4, and anti‐CD8, and anti‐IFNγ antibody were used. Details seen in Supporting Information.

### 4.16 RT‐qPCR Analysis

RNA from colon and muscle tissues of mice were extracted with RNAiso plus. RT‐qPCR was performed following previous protocols (Liu et al. 2024). The sequence of used primers was shown (Table S5).

### 4.17 Western Blot Analysis

Total protein from colon or muscle tissues of mice, and muscle nuclear protein was extracted. Primary and secondary antibodies were used. Details seen in Supporting Information.

### 4.18 Statistical Analysis

All data were expressed as mean ± standard error of mean (SEM), quartiles, or case number combined with percentage. Unpaired student's *t*‐test, Chi‐squared test, Wilcoxon, and Kruskal–Wallis or One‐way ANOVA tests with post hoc analysis (Bonferroni test used in groups with different sample size, Tukey's test used for the same sample size, and Wilcoxon and Dunn tests for non‐parametric tests) were performed using SPSS and GraphPad. Beta diversity of the gut microbiota at different levels was performed by permutational multivariate ANOVA (PERMANOVA). LDA was executed to detect the differential microbiota, and LDA scores (log 10) > 2 for human, > 3 for mice of FMT study were shown. Metastats analysis was also used to find differences of microbes utilizing the permutation test to get *p* values of each taxonomy between groups, and then Benjamini and Hochberg False Discovery Rate (FDR) was calculated to adjust the *p* value and obtain the *q* value. Percentage of bacteria associated with muscles amongst all bacteria at different taxa levels was shown. Spearman analysis was performed to detect the correlation between muscle indicators (ASMI and grip strength were adjusted by gender) or physiological conditions (age, BMI, gender) and gut microbiota relative abundance. Predicted functions of the microbiota between groups were executed by PICRUSt2 software. OPLS‐DA was used to display the differences of microbial metabolites between people with or without sarcopenia, and probiotic medium and supernatant. The model interpretation rate and predictive ability were evaluated by using R^2^X, R^2^Y and Q^2^. VIP > 1 from OPLS‐DA results, *p* value (< 0.05), and absolute Log_2_FC (> 1.0) were utilized to determine the differential metabolites. Biological pathways of top 50 differential metabolites were annotated by MSEA. ROC curve was analyzed to distinguish people with or without sarcopenia based on differential metabolites. Sensitivity, specificity, and AUC with 95% confidence interval (CI) were shown. The top 10 KEGG annotation of each treated group was shown based on differential genes from RNA‐seq compared to the control group (*p* < 0.05). Fold enrichment and *q* value were shown. R software was also used for analyses and visualization. *p* or *q* value < 0.05 were regarded as significantly different. The source of all equipment and materials was shown in Table S6.

## Author Contributions

R.M.Y.W., and J.J.Y.S.: conceptualization; C.L., N.B., S.H.W., J.Y., M.I., W.H.C., C.B., and G.D.: methodology; C.L., P.Y.W., N.B., and B.L.: investigation; C.L., and H.Y.W.: visualization; R.M.Y.W., N.Z., S.K.H.C., W.H.C., and G.D.: project administration; R.M.Y.W., J.Y., and M.I.: supervision; C.L.: writing – original draft; all authors: writing – review and editing; R.M.Y.W., and W.H.C.: funding acquisition. All authors have directly accessed and verified the underlying data reported in the manuscript.

## Ethics Statement

All human and animal studies have been approved by ethics committee, and conducted according to the 1964 declaration of Helsinki. The patient consents were obtained prior to inclusion in the study.

## Conflicts of Interest

The authors declare no conflicts of interest.

## Supporting information


Data S1.

## Data Availability

The raw data of 16S rDNA sequencing and transcriptome sequencing data of mouse, and human metagenomic sequencing data have been deposited in the NCBI Sequence Read Archive database (PRJNA1045861) under the link (https://www.ncbi.nlm.nih.gov/bioproject/?term=PRJNA1045861). The metabolomics data are available in Metabolomics Workbench repository (PR001868) with study ID ST002998 (https://dev.metabolomicsworkbench.org:22222/data/DRCCMetadata.php?Mode=Study&StudyID=ST002998&StudyType=MS&ResultType=1) and ST003002. (https://dev.metabolomicsworkbench.org:22222/data/DRCCMetadata.php?Mode=Study&StudyID=ST003002).
