# Supplementary material for: From Clinical to Benchside: Lacticaseibacillus and Faecalibacterium Are Positively Associated With Muscle Health and Alleviate Age‐Related Muscle Disorder
Source: Aging Cell. 2025 Jan 19;24(5):e14485. doi: 10.1111/acel.14485 (PMC12073917; doi:10.1111/acel.14485)
Supplement: Supplementary file 1 — Data S1. [file ACEL-24-e14485-s001.docx]

**Supporting information**

**From clinical to benchside: *Lacticaseibacillus* and *Faecalibacterium* are positively associated with muscle health and alleviate age-related muscle disorder**

**Supplementary method**

**1.1 Participant recruitment**

Inclusion criteria were 1) 60 years old or above, 2) live in Hong Kong in the last 3 years, 3) Chinese subjects only. Exclusion criteria were 1) use of antibiotics, probiotics, or prebiotics in the last 2 months, 2) chairbound, or bedbound, 3) cognitive dysfunction with severe dementia, stroke with paralysis, infectious disease, cancer, severe gastrointestinal (GI) tract disease or gastrointestinal surgery history, chronic kidney disease and undergoing dialysis, severe active rheumatoid arthritis (RA), 4) pathological fracture e.g., malignancy, and 5) non-pathological fracture within 1 year.

The AWGS 2019 consensus was used for sarcopenia diagnosis. Briefly, bioelectrical impedance analysis (BIA) was performed to calculate the skeletal muscle mass index (SMI) (skeletal muscle mass/height^2^). The previous published prediction model was utilized to predict ASMI according to dual energy x-ray absorptiometry (DXA) analysis (R^2^ = 0.862) (Liu et al., 2023). This is because BIA overestimated the muscle index (2.89 ± 0.38 kg/m^2^ higher) compared to DXA. Cut-off points of ASMI, handgrip strength, 6-metre walk test, 5-time chair stand test for sarcopenia diagnosis were shown in Figure S7. Participants with low ASMI combined with either low handgrip strength or poor performance were diagnosed as sarcopenic.

**1.2 Questionnaire assessments**

Physical Activity Scale for the Elderly (PASE) is a 12-item self-reported physical activity questionnaire for old people (Vaughan et al., 2013). Participants were asked to record the frequency and duration of different types of physical activities in their leisure, work, and household time over the past 7 days. Charlson Comorbidity Index (CCI) containing 19 items of medical comorbid conditions, such as myocardial infarction, cognitive heart failure, peripheral vascular disease, dementia, cerebrovascular disease, chronic lung disease, and diabetes was used (Charlson et al., 2022). Bristol Stool Scale (BSS) was utilized for human stool form category (Vandeputte et al., 2016). 7 groups from hardest to softest were classified to predict stool health, such as constipation, healthy, lacking fibre, or diarrhea. Education, smoking, alcohol drinking, dietary habit, fracture and surgery history, and medical history were investigated in the self-reported questionnaire. The food weight, calories, protein, carbohydrates, fibre, sugar, fat, vitamins, and other micronutrients were measured using validated local food frequency questionnaires and analysed by ESHA CLOUD SERVICES Nutrition Analysis Software (Food Processor^®^) (Liu et al., 2022).

**1.3 Shotgun metagenomic analysis**

Participants brought fresh stool samples within 2 hours and preserved at -80 °C until use. DNA extraction from the stool samples was performed using the Magnetic Soil and Stool DNA Kit following the manufacturer’s protocols. The library was constructed using NEBNext® Ultra™ DNA Library Prep Kit for Illumina. 1μg DNA of each sample was fragmented to a size of 350 bp using sonication. After PCR amplification and purification, the distribution size of library was analysed and quantified using Agilent2100 Bioanalyser and real-time PCR, respectively. cBot Cluster Generation System was used for index-coded sample cluster. The library preparations were sequenced on the Illumina NovaSeq 6000 platform to generate paired-end reads. The number of reads was approximately 70 million per sample. The filtered reads were assembled into metagenomic contigs with SOAPdenovo software. MetaGeneMark software and CD-HIT software were used for gene prediction and abundance analysis of faecal microbiota. Host sequences were excluded from the dataset by Bowtie2. The gene abundance of each sample was calculated based on the number of mapped reads and the length of each gene. Relative abundance was calculated as the percentage of each bacterium among all bacteria at different taxonomic levels. The taxonomy prediction was based on DIAMOND software.

**1.4 Gavage solution and antibiotic cocktails preparation for FMT**

After insoluble substance removal via centrifuging at 800 g for 3 min, the stool solution was subpackaged in sterile tubes (the amount is based on a single gavage) and stored in -80 ℃ until use. Antibiotic cocktails were added (0.1 g/L vancomycin, 0.2 g/L ampicillin, neomycin, and metronidazole) into drinking water daily for 2 weeks to deplete the gut microbiota (Wong et al., 2017).

**1.5 Probiotic preparation**

*Lacticaseibacillus rhamnosus* ATCC 53103 (LR) was cultured on MRS Agar. A single colony of LR was cultured in DeMan-Rogosa-Sharpe broth to obtain 10^8^ colony forming unit (CFU)/mL in sterile water in the anaerobic chamber. *Faecalibacterium prausnitzii* ATCC 27768 was cultured on freshly prepared pre-reduced YBHA media. A single colony from the YBHA plate was cultured in the YBHI media to obtain 10^8^ CFU/mL. FP was confirmed by 16S ribosomal DNA gene sequencing, and LR was confirmed by MALDI-TOF with a score above 2. For the preparation of the mixed inoculum of LR and FP, the respective cultured bacteria were mixed at 1:1 ratio. The gram staining was performed following protocols, and the LeicaDM1000 LED microscope was used. After 18 h probiotic incubation, probiotic culture medium or supernatant of the culture was filtered and put in -80 ℃ freezer for further metabolomic analysis.

**1.6 DNA extraction and 16S rDNA sequencing**

The genomic DNA was extracted from mouse stools using the E.Z.N.A. ®Stool DNA Kit as per the manufacturer’s instructions. The total DNA was diluted in by Elution buffer. PCR products were purified via AMPure XT beads and quantified by Qubit. Agilent 2100 Bioanalyser was used to assess the size of amplicon library, and the Library Quantification Kit for Illumina was used to evaluate the quantity. The 16S rDNA sequencing was conducted on the NovaSeq PE250 platform. After quality control (QC), dereplication by DADA2, and sequence alignment by Blast, feature sequences were annotated with SILVA database for each representative sequence. According to the count of sequence, normalized count by percentage was used as relative abundance.

**1.7 LC-MS and GC-MS analyses**

20 mg stool samples from each participant were added with 400 μL solution (methanol: water = 7:3 V/V) with internal standard to prepare for metabolomic analysis. The LC-MS system (1290 Infinity LC UPLC coupled to 6545 Q-TOF MS, Agilent, USA) was utilized. The analysis was conducted using a Waters ACQUITY UPLC HSS T3 C18 column (1.8 µm, 2.1 mm*100 mm). The original data file was transformed into the mzML format by ProteoWizard software. The XCMS program was used to execute the peak extraction, peak alignment, and retention time correction. After the correction, the comprehensive metabolic identification information was obtained from the MetWare self-built database, public database, AI database, as well as metDNA. Probiotic supernatant samples were centrifuged and performed LC-MS/MS analyses by using an UHPLC system with a UPLC BEH Amide column coupled to Orbitrap Exploris 120 mass spectrometer. Parameter setting of mass spectrometer was as previous published methods (Sui et al., 2022). The peak detection, extraction, alignment, and integration were obtained and annotated. For GC-MS analysis, 20 mg human stool sample was mixed with 1 mL phosphoric acid (0.5% V/V) solution to prepare for SCFAs analysis. Agilent 7890B gas chromatograph coupled to a 7000D mass spectrometer were used. GC column DB-FFAP and helium carrier gas were used for analysis. The multiple reaction monitoring mode was used to analyse all samples. The medium and supernatant of probiotics (LR and FP) were obtained from three separate preparations (*n* = 3 per group). 100 μL of each sample was added with 400 μL extract solution containing standard mixture (methanol: acetonitrile = 1: 1) to prepare for metabolomic analysis.

**1.8 Muscle functional tests**

Grip strength and *ex-vivo* muscle functional test was conducted following the previous protocol (Wang et al., 2020). Briefly, mouse forelimb grip strength was performed three times on the force gauge, and the highest value was used for analysis. Since gastrocnemius mainly contains fast twitch fibres and plays important roles in body movements, fresh gastrocnemius was isolated from the right limb of anesthetized mice for *ex-vivo* functional test (Figure S6c). After fixation to the dynamic muscle system and stabilization for 15 min in the Ringer solution, muscle was activated by two tetanic contractions. Continuous stimulations were conducted to decide the optimal muscle length. Twitch and tetanic (80 Hz for 300 ms) force were measured at the optimal length three times for each. The average value was utilized for analysis. For the fatigue assessment, the muscle received a series of tetanic stimuli at 150 Hz. Stimuli were executed at intervals of 10s for 600s. Catwalk was performed to evaluate the gait performance of aged mice. Mice were trained once a week before the formal test. Three successful trials of illuminated walkway crossing were utilized to obtain gait parameters. Average body speed, forelimb and hindlimb swing speed were recorded. For the endurance test, treadmill exercise was executed as previously reported (Klein et al., 2021). After three times training (10 min at 17 cm/s) before the formal test, mice received a running protocol with 10 min at 10 cm/s, followed by 40 min at 29 cm/s, and then the speed was gradually increased by 1 cm/s. Mice were defined as exhibiting exhaustion if they retreated to the grid three times within 30 s. Distance of the endurance test was calculated and recorded. To observe the voluntary activity, mice were placed in a square white box for 10 min. The movement was recorded by an overhead camera, and the video was analysed by ANY-maze Video Tracking Software. A central zone was defined as the centre of the field. The total distance travelled within 10 min was recorded.

**1.9 Immunofluorescence staining**

The gastrocnemius of the left leg of mice was isolated and washed in cold PBS. Samples were dehydrated and sectioned at 8 μm thickness under -20℃ by Cryostar NX70 microtome. Myosin heavy chain (MHC) staining with anti-MHC I, anti-MHC IIa, anti-MHC IIb, and Laminin staining with anti-Laminin 2 alpha antibody were performed. The myofibre type and myofibre cross-sectional area (CSA) were analysed, respectively. The fluorescence light microscope system with 200× magnification was utilized for visualization of stained slides and image capture. Image J (NIH, MD, USA) was used for image analysis.

The colon was isolated from the mouse abdomen and washed in cold PBS. Paraffin-embedded sections (4μm) were prepared, and Hematoxylin and Eosin (H&E) staining were performed following protocols. After deparaffinization, rehydration, and antigen retrieval by heated citric acid of colon sections, anti-Muc2 antibody was used for Muc2-positive cells identification.

**1.10 Transcriptome sequencing**

Total RNA extraction from TA of aged mice was performed using Trizol reagent according to the instruction of the manufacturer. Purified mRNA by Dynabeads Oligo (dT) was fragmented using the Magnesium RNA Fragmentation Module. cDNA reverse-transcribed from cleaved RNA fragments was used to establish the cDNA library. The sequencing procedure was executed on the Illumina Novaseq 6000 platform. Cutadapt and HISAT2 package were used to filter and align reads to corresponding genome. StringTie, gffcompare, and ballgown were used for gene abundance quantification based on FPKM.

**1.11 Mitochondria density assessment**

The mice gastrocnemius was diced, and fixed in glutaraldehyde, washed in Sorensen’s phosphate buffer, immersed in osmium tetroxide, dehydrated by graded ethyl alcohols, and washed by propylene oxide. Epoxy embedding medium with or without propylene oxide was used. Resin blocks were sectioned by Leica EM UC7 Ultramicrotome and Uranyl acetate staining procedure was performed. Transmission electron microscope (TEM) was used for the image capture of mitochondria in muscle tissue.

**1.12 Flow cytometry of colon T cells**

Fresh colon tissues were prepared for T cell detection. 1 mM Dithiothreitol (DTT) in PBS and 30 mM EDTA in PBS were used to isolate the lamina propria of colon as previous methods. 0.5 mg/mL Collagenase Ⅳ and 0.25 mg/mL DNaseⅠ in RPMI 1640 medium with 10% FBS was used to digest the diced tissue with agitation. The digested tissues were filtered by a 70 μm strainer. Isolated colon cells were obtained, washed, and resuspended by the medium with cell stimulation cocktail. The isolated cells were cultured in the dish for 4 h at 37℃. After cells were transferred to tubes and centrifugated, the Fc receptor binding inhibitor antibodies were added for 20 min. Surface antibodies anti-CD3, anti-CD4, and anti-CD8 were diluted by flow cytometry staining (FCS) buffer and incubated. After washed by FCS buffer, the Foxp3/Transcription Factor Staining Buffer Set was used following the manufacturer's procedure to fix cells and increase the permeability of cells for further nuclear protein staining. Anti-interferon gamma (IFNγ) antibody was diluted in permeabilization buffer and used for incubation as previously. After washing, samples were added with FCS buffer and analysed with BD FACSAria Fusion Flow Cytometry Cell Sorter. Flowjo (V. 10, Treestar) was used for data analysis.

**1.13 Western blot analysis**

Total protein from colon or muscle tissues of mice was extracted by RIPA buffer with protease/phosphatase inhibitor cocktail, and quantified by Pierce BCA Protein Assay Kit. Protein extracts were transferred to polyvinylidene fluoride (PVDF) membranes after the sodium dodecyl sulfate (SDS)-polyacrylamide gels electrophoresis. For the muscle nuclear protein extraction, we followed the previous published method (Cox et al., 2006). 250-STMDPS buffer and NE buffer were used. The western blot procedure was similar. As for colon, primary antibodies were anti-Claudin-1 (1:2000), anti-Occludin (1:2000), anti-E-cadherin (1:2000). As for muscle, anti-optic atrophy-1 (OPA1), anti-dynamin related protein 1 (DRP1), anti-mitofusin 1 (MFN1), anti- mitofusin 2 (MFN2), anti-carnitine palmitoyl transferase 1A (CPT1A), anti-ATP synthase lipid-binding protein (ATP5A1), anti-cytochrome c oxidase IV (COX IV), anti-nuclear respiratory factor (NRF1), anti-peroxisome proliferator-activated receptor gamma coactivator 1α (PGC1α), anti-forkhead box o 3a (FoxO3a), anti-phospho-FoxO3a (Ser253), anti-Fbxo32 (known as Atrogin1), and anti-Trim63 (known as Murf1) were 1:2000 used. Anti-β-actin (1:10000), anti-GAPDH (1:5000), and anti-Histone H3 (1:2000) antibodies were also used as internal control. Secondary antibodies (1:5000) were anti-rabbit IgG, HRP-linked antibody, and goat anti-mouse IgG (H + L) antibody.

**1.14 Quality control of sequencing data**

Quality control (QC) of all sequencing data was shown. As for shotgun metagenomic sequencing, degradation degree and potential contamination of DNA were monitored on 1% agarose gels, and Qubit® dsDNA Assay Kit was used to measure the DNA concentration in Qubit® 2.0 Flurometer (Life Technologies, CA, USA). OD value between 1.8 – 2.0, and DNA contents above 1ug were used to construct library. QC of 16s rDNA sequencing was performed by fqtrim software (v0.94) (parameters: '-P 33 -w 100 -q 20 -l 100 -m 5 -p 1 -V -o trim.fastq.gz') and Vsearch (v2.3.4) for chimeric filtering. Total ions current (TIC) overlapping map of QC and Pearson correlation analysis of QC were used for LC-MS QC of human stool samples, and those with high signal stability and correlation coefficient were used for metabolomic analysis. The probiotic supernatant and culture medium QC of LC-MS was based on deviation filtering by coefficient of variation, single peak filtering, missing value recording, normalization using internal standard. For RNA-seq, after removing reads containing adapters, polyA and polyG, or more than 5% of unknown nucleotides (N), and low quality reads containing more than 20% of low quality (Q-value≤20) bases. FastQC (0.11.9) was used to verify the sequence quality. Samples with high QC were included for further analysis.


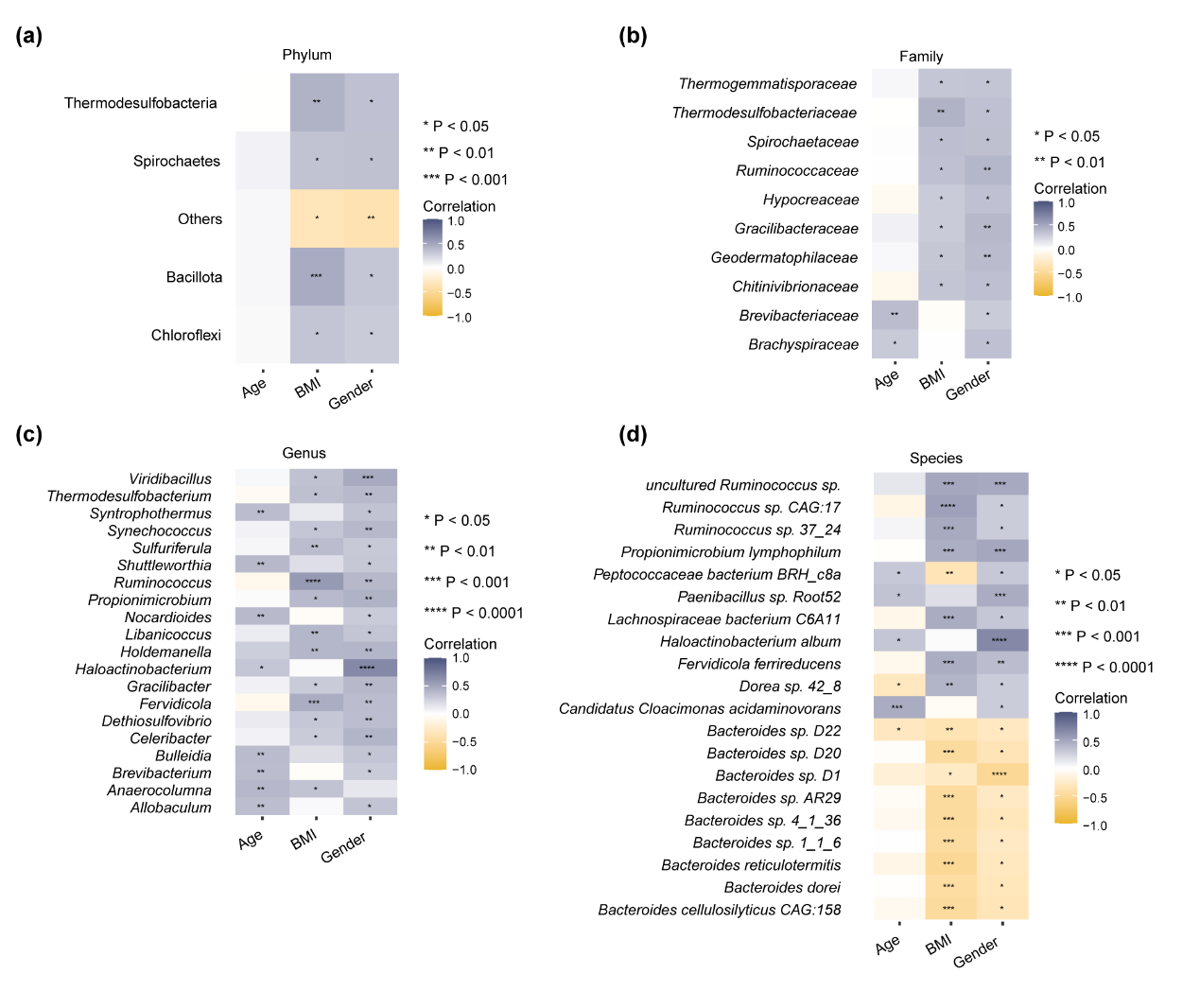


**Figure S1** Correlation of gut microbiota abundance and physiological conditions (Age, BMI, Gender) (*n* = 51). (a) Top 5 gut microbiota at the phylum level that related to at least 2 physiological conditions. (b) Top 10 gut microbiota at the family level that related to at least 2 physiological conditions. (c) Top 20 gut microbiota at the genus level that related to at least 2 physiological conditions. (d) Top 20 gut microbiota at the species level that related to at least 2 physiological conditions. ^*^*P* < 0.05, ^**^*P* < 0.01, ^***^*P* < 0.001, ^****^*P* < 0.0001, by Spearman correlation analysis. Gender: female = 0, male = 1.


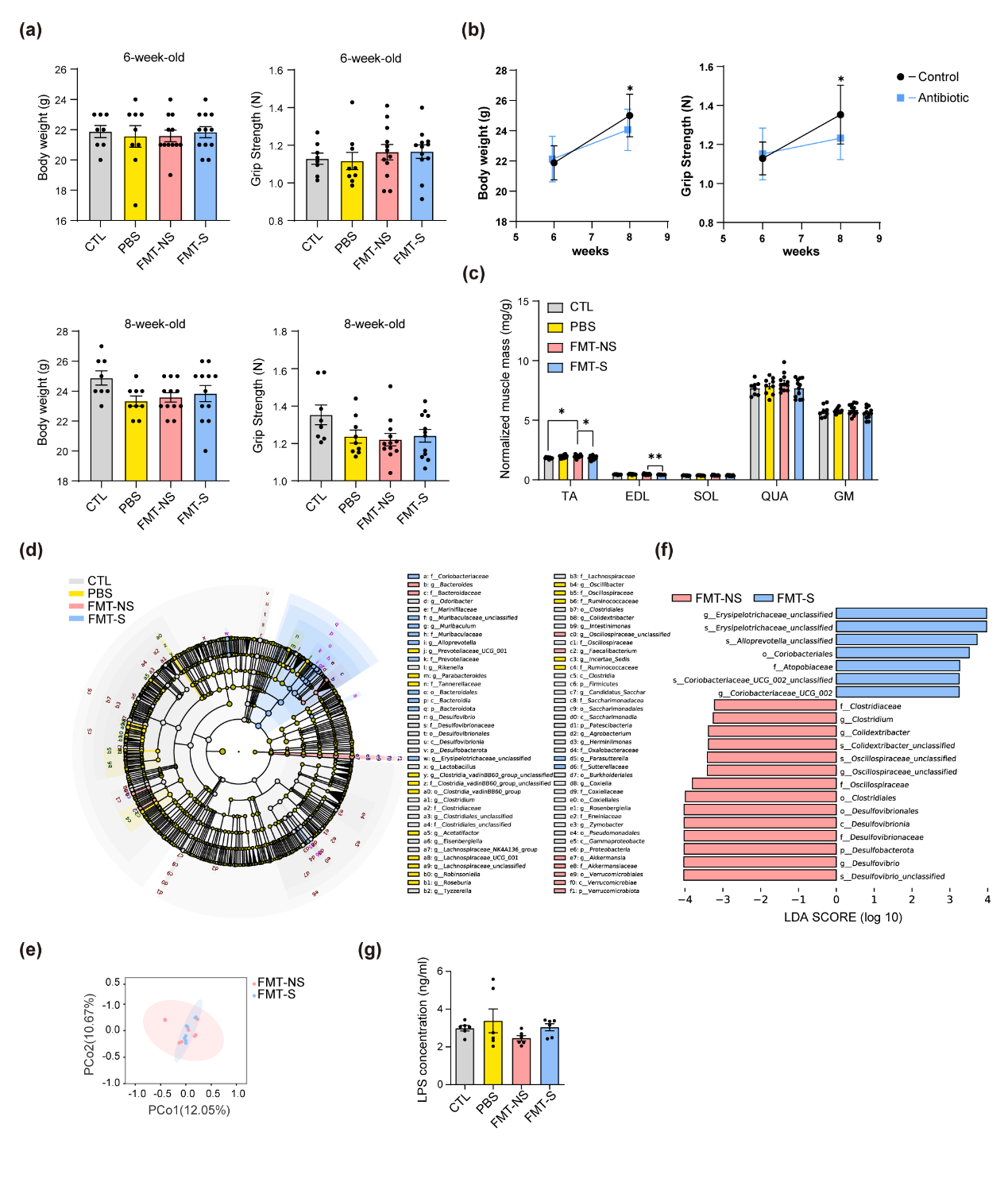


**Figure S2** Mice conditions of FMT study at baseline, after antibiotic treatment, and normalized muscle mass, gut microbiota composition, and LPS concentration after FMT treatments. (a) Body weight and forelimb grip strength at baseline (6-week-old), and after antibiotic treatment (8-week-old) amongst groups (*n* = 8 to 12), by ANOVA followed by Bonferroni test. (b) Comparison of body weight and forelimb grip strength between mice with (*n* = 33) or without (*n* = 8) antibiotic cocktails. ^*^*P* < 0.05, by two-tailed, unpaired student’s t test. (c) Normalized muscle mass by body weight amongst groups (*n* = 8 to 12). Data are shown as means ± SEM (error bars). ^*^*P* < 0.05, ^**^*P* < 0.01, by ANOVA followed by Bonferroni test. (d) Cladogram plot of LEfSe based on relative abundance of gut microbiota amongst 4 groups. Linear discriminant analysis (LDA) score (log 10) > 3, *P* < 0.05 by Kruskal-Wallis followed by Wilcoxon test was shown (*n* = 6 to 8). (e) PCoA of gut microbiota between two FMT groups based on Jaccard distances (*n* = 8). (f) LEfSe analysis of relative abundance of gut microbiota between FMT groups, LDA score (log 10) > 3, *P* < 0.05 by Wilcoxon test was shown (*n* = 8). (g) Serum concentration of LPS amongst CTL, PBS, and FMT groups (*n* = 6), by ANOVA followed by Bonferroni test.


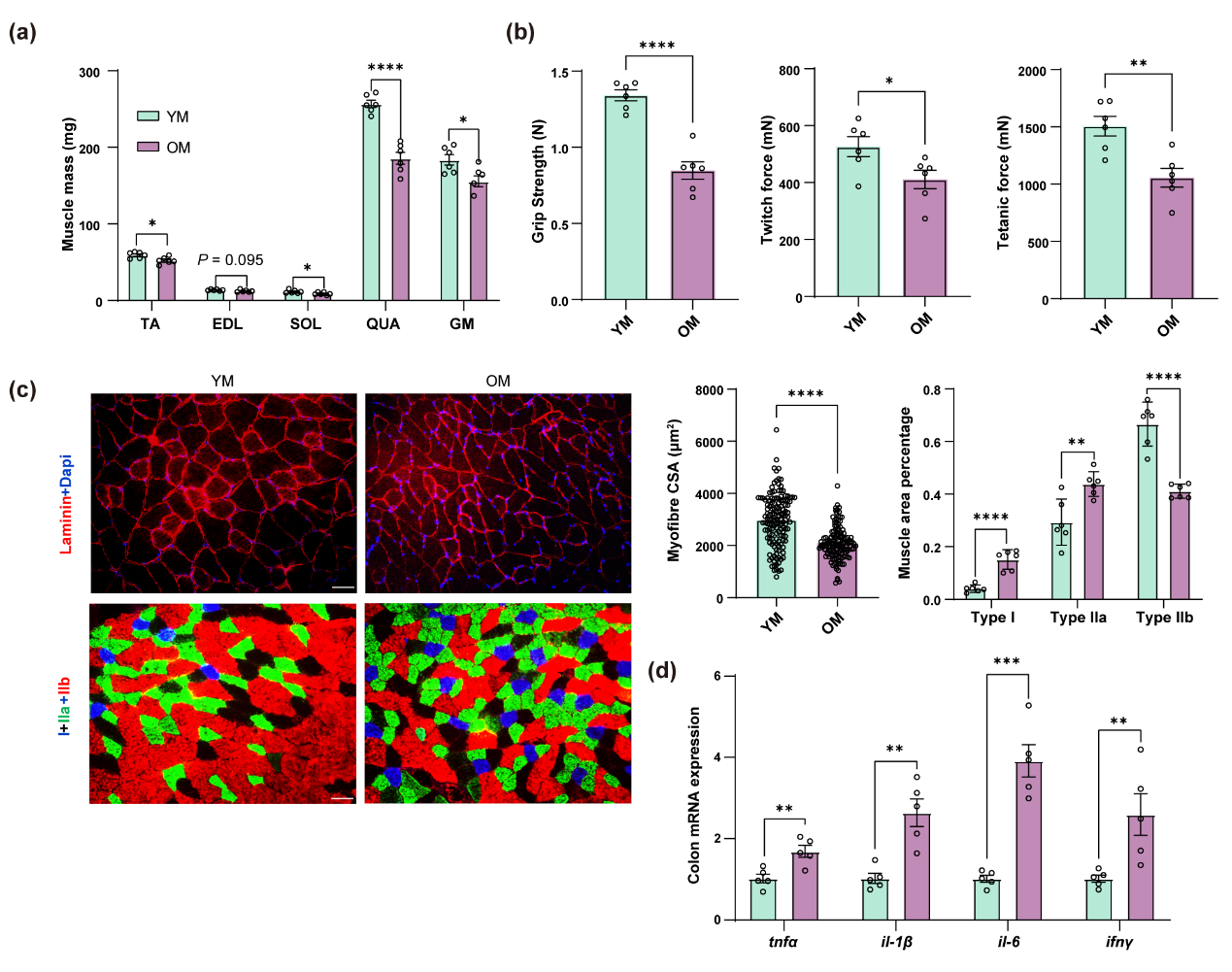


**Figure S3** Muscle and colon status of old mice (OM) compared to young mice (YM). (a) Lower limb muscle mass (*n* = 6). (b) Forelimb grip strength, and twitch and tetanic force of gastrocnemius (*n* = 6). (c) Immunofluorescence staining of gastrocnemius Laminin and MHC (scale bar = 50 μm). Myofibre CSA and the fibre size distribution (*n* = 3 mice per group, 141 myofibres per group). Muscle area percentage of different fibre types (*n* = 3 mice, 2 positions of MHC staining per group). (d) Colon mRNA expression of pro-inflammatory markers (*n* = 5 mice per group). Data are shown as means ± SEM (error bars). ^*^*P* < 0.05, ^**^*P* < 0.01, ^***^*P* < 0.001, ^****^*P* < 0.0001, by two-tailed, unpaired student’s t test.


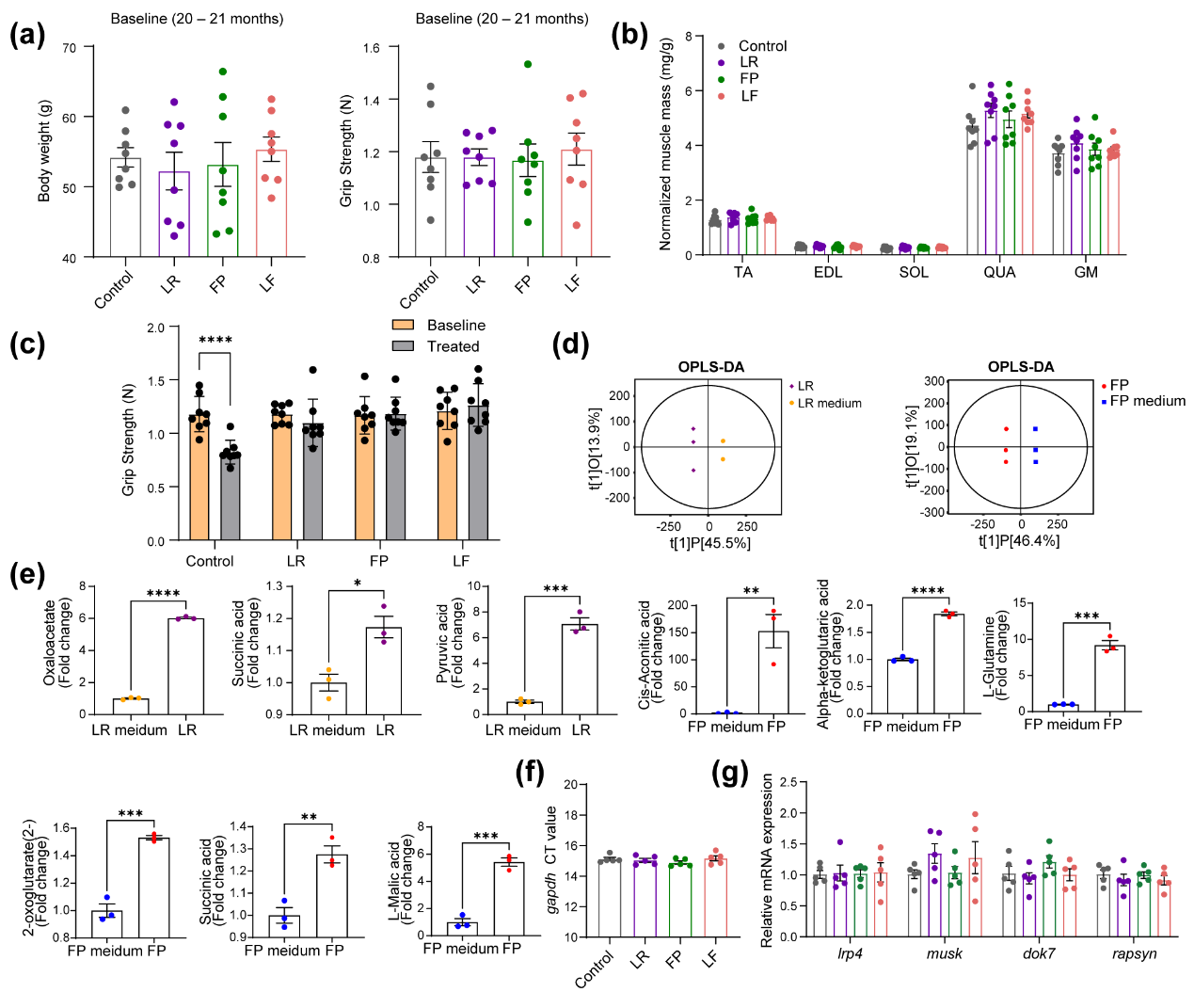


**Figure S4** Baseline of aged mice, normalized muscle mass after probiotic treatments, grip strength before and after probiotic treatments, TCA cycle-related metabolites secreted by LR and FP, *gapdh* CT value, and expression of neuromuscular junction (NMJ)-related genes after probiotic treatments. (a) Body weight and forelimb grip strength at baseline of aged mice (20 – 21 months). (b) Normalized muscle mass by body weight amongst groups, by ANOVA followed by Tukey’s post hoc test. (c) Grip strength comparisons before and after treatments. (d) OPLS-DA plot derived from probiotic supernatant and culture medium (*n* = 3 per group). (e) Elevated TCA cycle substances secreted by LR or FP (*n* = 3). ^*^*P* < 0.05, ^**^*P* < 0.01, ^***^*P* < 0.001, ^****^*P* < 0.0001, by two-tailed, unpaired student’s t test (c, e). (f) CT value of *gapdh* amongst control and probiotic groups. (g) Expression of NMJ-related *low-density lipoprotein receptor-related protein 4* (*lrp4*), *muscle associated receptor tyrosine kinase* (*musk*), *docking protein 7* (*dok7*), and *receptor associated protein of the synapse* (*rapsyn*) at mRNA levels in EDL muscles (*n* = 5), by ANOVA followed by Tukey’s post hoc test (a, b, f, g). Data are shown as means ± SEM (error bars).


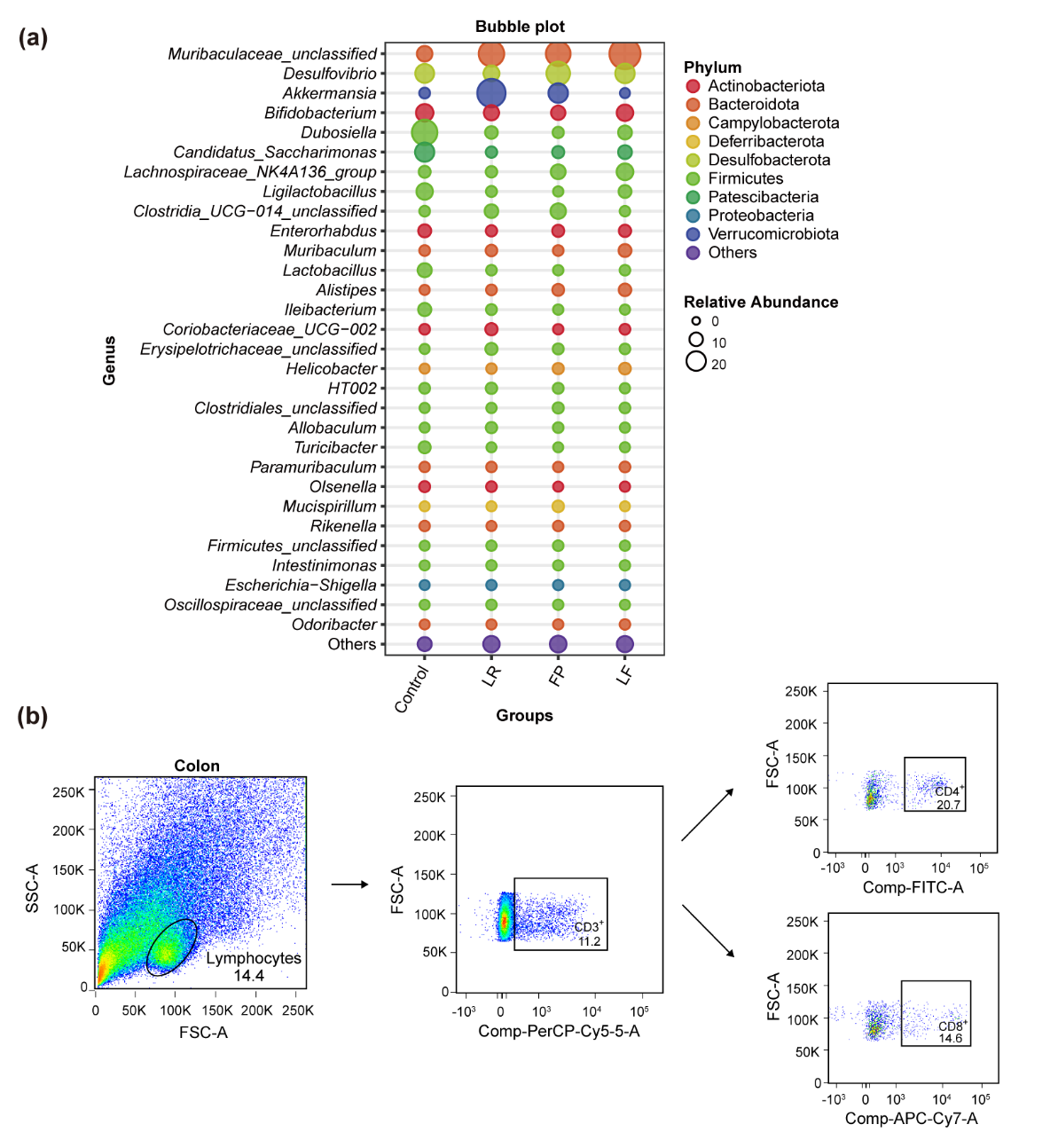


**Figure S5** Gut microbiota alterations after probiotic treatments, and colon tissue flow cytometry gating. (a) Bubble plot of higher abundant gut microbes at genus levels among groups (*n* = 5 per group). (b) Flow cytometry gating of CD3^+^ CD4^+^ T cells and CD3^+^ CD8^+^ T cells.


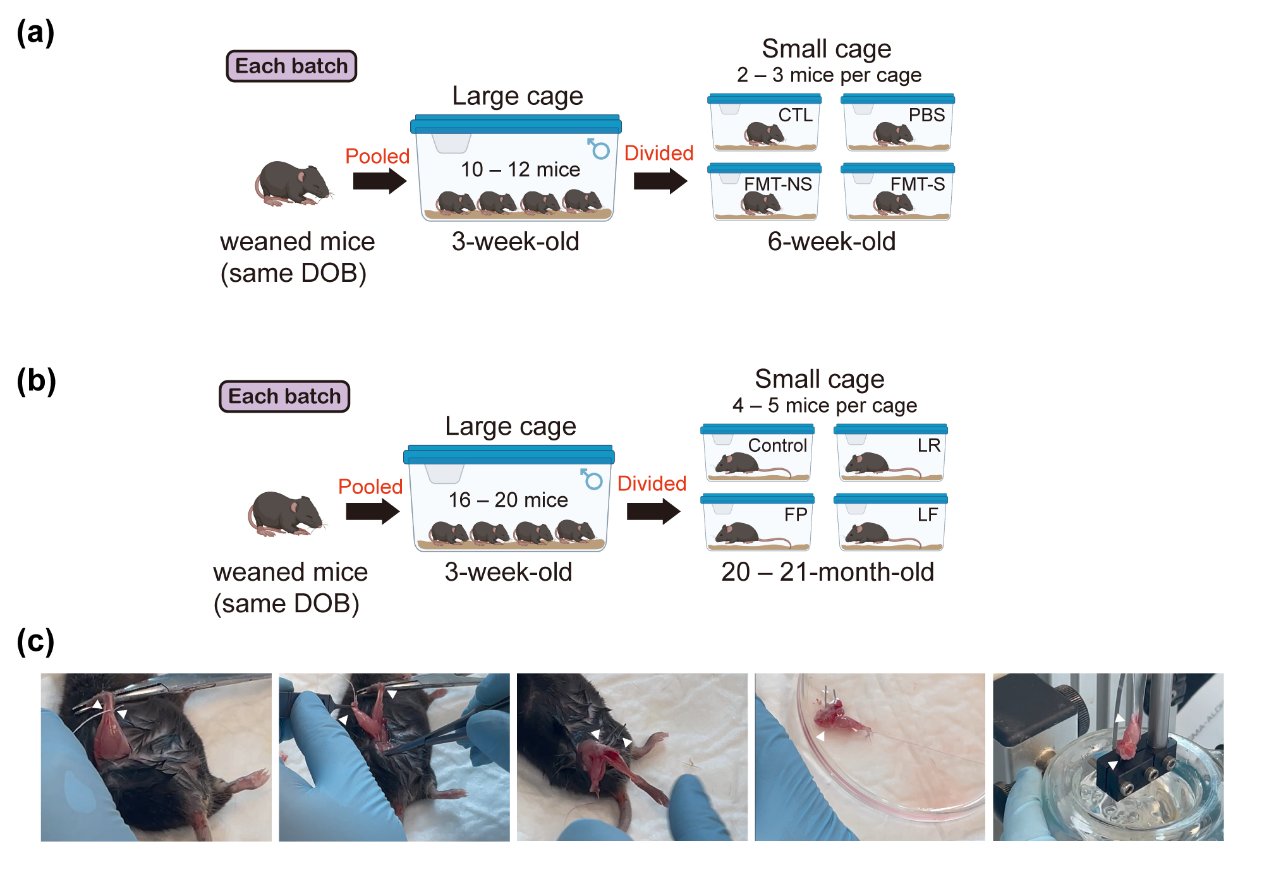


**Figure S6** Mouse caging strategies, and gastrocnemius isolation for *ex-vivo* functional test. (a) Weaned mice with the same date of birth (DOB) were pooled at 3 weeks old and divided into small cages for different treatments at 6 weeks old for the FMT study as one batch. (b) Weaned mice with the same DOB were pooled at 3-weeks-old (considering natural mortality rate) and divided into small cages for different treatments at 20–21 months old for the probiotic study as one batch. (c) For the *ex-vivo* functional test, the right Achilles tendon was tied with an inelastic string. The right gastrocnemius was carefully isolated together with the Achilles tendon, and a U-shape iron wire was passed through the knee space. To avoid the damage of gastrocnemius heads, half of the femur was chosen as the cut-off position. The isolated gastrocnemius was washed and removed the surrounding fat and fascia, and then fixed to the machine (force transducer side with the string, the immersion side with the iron wire) and immersed with Ringer solution.


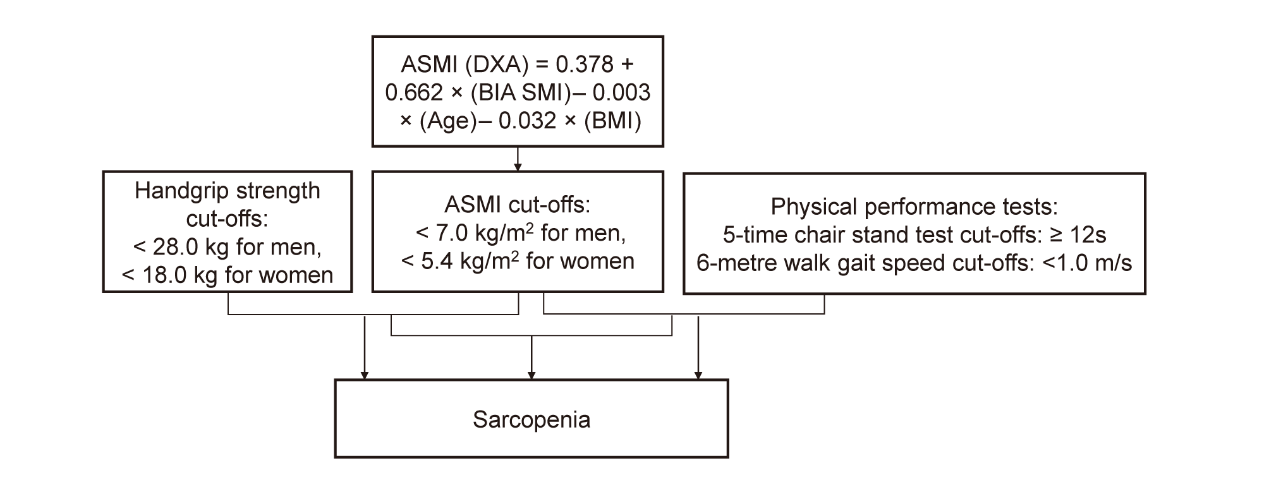


**Figure S7** Diagnosis of old people with or without sarcopenia. Predicted ASMI was performed based on BIA data and the previous prediction model, old people with lower ASMI, and lower handgrip strength or poor physical performance were diagnosed with sarcopenia. DXA, dual energy x-ray absorptiometry; BIA, Bioelectrical impedance analysis; SMI, skeletal muscle mass index; BMI, body mass index; ASMI, appendicular skeletal muscle mass index.

**Table S1** Demographic information of old people with or without sarcopenia for gut microbiota analysis

|  | **Non-sarcopenia** | **Sarcopenia** | ***P* value** |
| --- | --- | --- | --- |
| N | 23 | 28 | NA |
| Age | 69±3.9 | 71±5.8 | 0.233 |
| Gender | 17F/6M | 21F/7M | 0.929 |
| Height | 157±9.3 | 154.8±7.4 | 0.318 |
| Weight | 59±10.4 | 52.4±11.3 | 0.036 |
| BMI | 23.9±3.2 | 21.7±3.6 | 0.032 |
| ASMI | 5.5±0.8 | 4.9±0.7 | 0.007 |
| Grip strength | 24.4±6.1 | 19.3±5.9 | 0.003 |
| Gait speed | 1.2±0.2 | 1.0±0.2 | 0.001 |
| 5-time chair stand test | 9.1±1.6 | 13.6±4.9 | <0.001 |
| Alcohol drinker (%) | 0 | 0 | NA |
| Ever-smoker (%) | 0 | 0 | NA |
| Education (≥high school, n, %) | 19 (82.6%) | 22 (78.6%) | 1.000 |
| PASE score | 29.1±26.9 | 34.7±39.5 | 0.894 |
| CCI score | 2.7±0.6 | 2.5±0.6 | 0.291 |
| Bristol Stool Scale (n, %) |  |  |  |
| 1–2 | 4 (17.4%) | 2 (7.1%) | 0.390 |
| 3–4 | 14 (60.9%) | 15 (53.6%) | 0.601 |
| 5–7 | 5 (21.7%) | 11 (39.3%) | 0.241 |
| Cholesterol-lowering medication (%) | 34.8% | 28.6% | 0.634 |
| Antihypertensive medication (%) | 30.4% | 17.9% | 0.292 |
| Antidiabetic medication (%) | 8.7% | 3.6% | 0.439 |

NA, not applicable; F, female; M, male; BMI, body mass index; ASMI, appendicular skeletal muscle mass index; PASE, physical activity scale for the elderly; CCI, Charlson comorbidity index.

**Table S2** Daily nutrient intake of people for gut microbiota analysis

|  | **Non-sarcopenia** | **Sarcopenia** | ***P* value** |
| --- | --- | --- | --- |
| Weight (g) | 1255.9±353.8 | 1350.6±321.4 | 0.322 |
| Calories (kcal) | 1537±320 | 1557.6±290.5 | 0.810 |
| Protein (g) | 92.2±22 | 88.1±21.1 | 0.500 |
| Carbohydrates (g) | 214.9±57.8 | 215±49.2 | 0.996 |
| Total dietary fibre (g)^*^ | 17.6±5.6 | 14.5±4.9 | 0.041 |
| Total soluble fibre (g)^*^ | 1.2±0.8 | 0.7±0.7 | 0.037 |
| Total sugars (g) | 43±15 | 42.3±20.7 | 0.900 |
| Other carbs (g) | 127.6±39.9 | 124.5±38.1 | 0.777 |
| Fat (g) | 34.7±12.2 | 38.7±12.7 | 0.251 |
| Water (g) | 775.9±234.8 | 884.6±275.6 | 0.141 |
| Vitamin A (mcg) | 557.3±331.4 | 474.2±216 | 0.287 |
| Carotenoid (mcg) | 592.3±617.8 | 491.2±301 | 0.449 |
| Retinol (mcg) | 204.8±118.9 | 193.5±96.9 | 0.711 |
| Beta-carotene (mcg) | 1846.8±923.3 | 1984±1270.6 | 0.668 |
| Vitamin B1 (mg) | 1.4±0.5 | 1.2±0.4 | 0.322 |
| Vitamin B2 (mg) | 1.6±0.6 | 1.5±0.6 | 0.722 |
| Vitamin B3 (mg) | 18.5±6.1 | 17.1±5 | 0.385 |
| Vitamin B6 (mg) | 1.5±0.4 | 1.4±0.3 | 0.356 |
| Vitamin B12 (mcg) | 2.8±0.7 | 3±1.5 | 0.665 |
| Biotin (mcg) | 15.7±9.3 | 17.7±8.9 | 0.433 |
| Vitamin C (mg) | 270.2±326.2 | 189.4±127 | 0.234 |
| Vitamin D (mcg) | 10.4±6.9 | 9.8±6 | 0.740 |
| Vitamin E (mg) | 2.8±1.1 | 3±1.8 | 0.596 |
| Folate (mcg) | 220.9±100.5 | 192.7±61.2 | 0.225 |
| Vitamin K (mcg) | 84.5±63.1 | 109±89.9 | 0.276 |
| Pantothenic Acid (mg) | 3.7±1 | 3.8±0.9 | 0.881 |
| Calcium (mg) | 633.6±218.3 | 607.7±225.7 | 0.681 |
| Chromium (mcg) | 4.6±2.6 | 4.6±2.4 | 0.981 |
| Copper (mg) | 0.8±0.2 | 0.9±0.3 | 0.265 |
| Fluoride (mg) | 0±0 | 0±0.1 | 0.576 |
| Iodine (mcg) | 30.8±17.1 | 39.6±18.2 | 0.082 |
| Iron (mg) | 18.3±9.8 | 17.2±9.2 | 0.657 |
| Magnesium (mg) | 194.4±55.3 | 201.6±68.3 | 0.683 |
| Manganese (mg) | 2.3±1 | 2.6±1.1 | 0.351 |
| Molybdenum (mcg) | 8.9±4.6 | 11.2±6 | 0.147 |
| Phosphorus (mg) | 935±221.1 | 941.1±239.9 | 0.927 |
| Potassium (mg) | 1747.5±556.3 | 1725±479.4 | 0.877 |
| Selenium (mcg) | 99.1±27.7 | 103.4±24.2 | 0.559 |
| Sodium (mg) | 2237±1535.8 | 2105.3±1130.8 | 0.726 |
| Zinc (mg) | 6.6±1.8 | 7±1.6 | 0.492 |
| Omega-3 fatty acids (g) | 1.7±1.1 | 1.8±1.1 | 0.884 |
| Omega-6 fatty acids (g) | 5.1±2.3 | 5.7±3.3 | 0.438 |
| Caffeine (mg) | 27.7±37.7 | 43.7±60.2 | 0.274 |
| Choline (mg) | 274.6±69.1 | 275.5±93.9 | 0.970 |

**Table S3** Demographic information of hip fracture patients

|  | **High ASMI** | **Low ASMI** | ***P* value** |
| --- | --- | --- | --- |
| N | 19 | 19 | NA |
| Age | 82.1±7.2 | 82.6±6.1 | 0.797 |
| Gender | 15F/4M | 15F/4M | 1.00 |
| Height | 151.8±8 | 152.4±7.6 | 0.814 |
| Weight | 53±9.8 | 47.5±15.8 | 0.115 |
| BMI | 23.2±4.8 | 20.2±4.8 | 0.072 |
| ASMI | 5.5±0.7 | 4.4±0.6 | <0.001 |

NA, not applicable; F, female; M, male; BMI, body mass index; ASMI, appendicular skeletal muscle mass index.

**Table S4** Differential gut microbial metabolites between people with or without sarcopenia

| **Compounds** | **Class I** | **VIP** | **Log_2_FC** | ***P* value** | **AUC (95% CI)** |
| --- | --- | --- | --- | --- | --- |
| Terephthalic Acid | Phenolic acids | 2.12 | -2.22 | 0.029 | 0.714 (0.567 - 0.862) |
| Caprolactam | Heterocyclic compounds | 1.46 | -1.45 | 0.044 | 0.635 (0.478 - 0.792) |
| Indole-2-carboxylic acid | Organic acid and its derivatives | 1.43 | -1.37 | 0.031 | 0.632 (0.473 - 0.791) |
| 8-Azaguanine | Nucleotide and its metabolites | 1.92 | -1.18 | 0.031 | 0.722 (0.575 - 0.869) |
| Sorbitol 6-phosphate | Carbohydrates and its metabolites | 1.29 | -1.06 | 0.013 | 0.658 (0.494 - 0.823) |
| Benzyl-dimethyl-[2-[2-(2-methyl-4-octylphenoxy)ethoxy]ethyl]azanium | Others | 1.61 | 1.01 | 0.025 | 0.682 (0.534 - 0.830) |
| Cyclopassifloic acid C | Organic acid and its derivatives | 2.32 | 1.04 | 0.001 | 0.747 (0.611 - 0.882) |
| 2'-Deoxyinosine | Nucleotide and its metabolites | 1.98 | 1.08 | 0.031 | 0.694 (0.541 - 0.847) |
| Quinoline-2-carboxylic acid | Organic acid and its derivatives | 1.81 | 1.08 | 0.01 | 0.703 (0.559 - 0.848) |
| Malonyl-L-carnitine | Nucleotide and its metabolites | 2.57 | 1.15 | < 0.0001 | 0.865 (0.762 - 0.968) |
| 3,3-Dimethylglutaric acid | Organic acid and its derivatives | 2.4 | 1.18 | 0.018 | 0.674 (0.525 - 0.823) |
| Esculetin | Heterocyclic compounds | 3.18 | 1.18 | 0.001 | 0.818 (0.698 - 0.938) |
| 2-Hydroxycinnamic acid | Organic acid and its derivatives | 1.76 | 1.19 | 0.042 | 0.675 (0.526 - 0.825) |
| 2,3-Diacetoxy-7,8-epoxy-24,29-dinor-1,3,5-friedelatriene-20-carboxylic acid | Benzene and substituted derivatives | 1.02 | 1.2 | 0.017 | 0.649 (0.494 - 0.804) |
| Acetylsalicylic acid | Organic acid and its derivatives | 3.06 | 1.2 | 0.002 | 0.806 (0.680 - 0.932) |
| Pentylenetetrazol | Others | 1.95 | 1.21 | 0.002 | 0.724 (0.583 - 0.865) |
| Mancinellin | Terpenoids | 1.42 | 1.22 | 0.022 | 0.652 (0.501 - 0.804) |
| Carbenoxolone | Benzene and substituted derivatives | 1.55 | 1.23 | 0.032 | 0.675 (0.524 - 0.826) |
| Linoleoyl ethanolamide | Alcohol and amines | 1.06 | 1.24 | 0.039 | 0.661 (0.509 - 0.814) |
| Guanine | Heterocyclic compounds | 2.21 | 1.27 | 0.03 | 0.761 (0.620 - 0.902) |
| Purine | Nucleotide and its metabolites | 3.64 | 1.27 | < 0.0001 | 0.935 (0.867 - 1.000) |
| 1-(2,3-Dihydro-6,7-dimethyl-1H-pyrrolizin-5-yl)-2-hydroxy-1-propanone | Aldehyde, ketones, esters | 1.4 | 1.3 | 0.032 | 0.661 (0.509 - 0.814) |
| HC Toxin | Heterocyclic compounds | 1.41 | 1.3 | 0.034 | 0.661 (0.510 - 0.813) |
| Ser-Arg-Phe-Lys | Amino acid and its metabolites | 1.15 | 1.3 | 0.033 | 0.632 (0.478 - 0.786) |
| Arg-Thr-Ala-Arg | Amino acid and its metabolites | 1.31 | 1.32 | 0.05 | 0.658 (0.507 - 0.810) |
| Tryptamine | Tryptamines, cholines, pigments | 1.56 | 1.34 | < 0.0001 | 0.795 (0.663 - 0.927) |
| His-Glu-Gln-Lys | Amino acid and its metabolites | 1.49 | 1.39 | 0.04 | 0.683 (0.534 - 0.832) |
| Miltefosine | Organic acid and its derivatives | 1.35 | 1.4 | 0.035 | 0.652 (0.499 - 0.805) |
| HS-10296 | Others | 1.33 | 1.41 | 0.044 | 0.669 (0.518 - 0.820) |
| Telmisartan | Benzene and substituted derivatives | 1.56 | 1.45 | 0.027 | 0.691 (0.542 - 0.840) |
| Alangicine | Benzene and substituted derivatives | 1.48 | 1.54 | 0.028 | 0.675 (0.525 - 0.826) |
| Ursodeoxycholic Acid | Bile acids | 1.4 | 1.58 | 0.026 | 0.716 (0.570 - 0.862) |
| Uridine 5'-monophosphate | Nucleotide and its metabolites | 2.69 | 1.59 | 0.047 | 0.755 (0.618 - 0.891) |
| Therafectin | Heterocyclic compounds | 1.64 | 1.67 | 0.003 | 0.736 (0.595 - 0.877) |
| Arg-Tyr-Gln-Lys | Amino acid and its metabolites | 1.91 | 1.68 | 0.026 | 0.714 (0.571 - 0.858) |
| 5-Hydroxyhexanoic Acid | Organic acid and its derivatives | 1.57 | 1.7 | 0.029 | 0.399 (0.240 - 0.558) |
| Prilocaine | Alcohol and amines | 2.4 | 2.41 | 0.011 | 0.762 (0.632 - 0.892) |

Log_2_FC < 0 indicated the metabolite is abundant in people without sarcopenia. VIP, variable importance for the projection; FC, fold change; AUC, area under the curve; CI, confidence interval.

**Table S5** Primer sequence for PCR

| **Gene Name** | **Sequence** |
| --- | --- |
| Atrogin1 Forward | CAGCTTCGTGAGCGACCTC |
| Atrogin1 Reverse | GGCAGTCGAGAAGTCCAGTC |
| Claudin-1 Forward | GGGGACAACATCGTGACCG |
| Claudin-1 Reverse | AGGAGTCGAAGACTTTGCACT |
| Dok7 Forward | ATGCTGGTCTACAAGGACAAATG |
| Dok7 Reverse | AGCTACTGTCACATGGAACCT |
| GAPDH Forward | AACGACCCCTTCATTGAC |
| GAPDH Reverse | TCCACGACATACTCAGCAC |
| IFNγ Forward | ATGAACGCTACACACTGCATC |
| IFNγ Reverse | CCATCCTTTTGCCAGTTCCTC |
| IL-10 Forward | GCTCTTACTGACTGGCATGAG |
| IL-10 Reverse | CGCAGCTCTAGGAGCATGTG |
| IL-1β Forward | GCAACTGTTCCTGAACTCAACT |
| IL-1β Reverse | ATCTTTTGGGGTCCGTCAACT |
| IL-6 Forward | TAGTCCTTCCTACCCCAATTTCC |
| IL-6 Reverse | TTGGTCCTTAGCCACTCCTTC |
| Lrp4 Forward | GCACACGGAATAGCCAGCA |
| Lrp4 Reverse | GGATACAGGTACATTCGCCAAG |
| Mstn Forward | AGTGGATCTAAATGAGGGCAGT |
| Mstn Reverse | GTTTCCAGGCGCAGCTTAC |
| Murf1 Forward | GTGTGAGGTGCCTACTTGCTC |
| Murf1 Reverse | GCTCAGTCTTCTGTCCTTGGA |
| Musk Forward | TACAGAGGGGAGGTGTGTGAT |
| Musk Reverse | TCCCGGTAGGAGGTGTTGAA |
| Myod1 Forward | CCACTCCGGGACATAGACTTG |
| Myod1 Reverse | AAAAGCGCAGGTCTGGTGAG |
| Myog Forward | GAGACATCCCCCTATTTCTACCA |
| Myog Reverse | GCTCAGTCCGCTCATAGCC |
| Occludin Forward | TTGAAAGTCCACCTCCTTACAGA |
| Occludin Reverse | CCGGATAAAAAGAGTACGCTGG |
| Pgc1α Forward | TATGGAGTGACATAGAGTGTGCT |
| Pgc1α Reverse | CCACTTCAATCCACCCAGAAAG |
| Rapsyn Forward | GGCAGGACCAGACAAAGCAA |
| Rapsyn Reverse | CGAGTGAGCTGTTACCAAGCA |
| Tfam Forward | ATTCCGAAGTGTTTTTCCAGCA |
| Tfam Reverse | TCTGAAAGTTTTGCATCTGGGT |
| TNFα Forward | CCCTCACACTCAGATCATCTTCT |
| TNFα Reverse | GCTACGACGTGGGCTACAG |
| ZO-1 Forward | GCCGCTAAGAGCACAGCAA |
| ZO-1 Reverse | TCCCCACTCTGAAAATGAGGA |
| Hypervariable region of the 16S rRNA Forward | GTGCCAGCMGCCGCGG |
| Hypervariable region of the 16S rRNA Reverse | CCGTCAATTCMTTTRAGTTT |

Abbreviations: IFNγ, interferon γ, IL-10, interleukin-10; IL-1β, interleukin-1β; interleukin-6, IL-6; Lrp4, lipoprotein receptor–related protein 4; Mstn, myostatin; Murf1, muscle ring finger 1; Musk, muscle-specific kinase; Myod1, myoblast determination protein 1; Myog, myogenin; Pgc1α, peroxisome proliferator-activated receptor gamma coactivator 1α; Tfam, mitochondrial transcription factor a; TNFα, tumour necrosis factor α; ZO-1, zonula occludens-1; rRNA, ribosomal RNA.

**Table S6** Materials and sources

| **Commercial Assays** | **Source** |
| --- | --- |
| LPS ELISA kit (human) | CSB-E09945h, CUSABIO |
| FABP2 ELISA kit (human) | RK01348, Abclonal |
| LPS ELISA kit (mouse) | CSB-E13066m, CUSABIO |
| Magnetic Soil and Stool DNA Kit | TIANGEN Biotech |
| NEBNext® Ultra™ DNA Library Prep Kit for Illumina | NEB, USA |
| E.Z.N.A. ®Stool DNA Kit | D4015, Omega |
| Library Quantification Kit for Illumina | Kapa Biosciences |
| ATP Assay Kit | ab8335, Abcam |
| NAD/NADH Assay Kit | ab65348, Abcam |
| PrimeScript RT Reagent Kit | TaKaRa Biotechnology |
| Pierce BCA Protein Assay Kit | Thermo Fisher Scientific |
| Foxp3/Transcription Factor Staining Buffer Set | Cat. 00-5523-00, Invitrogen |
| Magnesium RNA Fragmentation Module | e6150, NEB |
| **Chemicals** |  |
| Prolong® diamond anti-fade mountant | Invitrogen |
| Phenylindole (DAPI) | Invitrogen |
| MRS Agar | Sigma |
| DeMan-Rogosa-Sharpe | Sigma |
| Trizol reagent | Invitrogen |
| Cell stimulation cocktail | Invitrogen |
| RNAiso plus | Takara |
| PowerUp SYBR Green PCR Master Mix | Thermo Fisher Scientific |
| protease/phosphatase inhibitor cocktail | Cell Signaling Technology |
| Dynabeads Oligo (dT) | Thermo Fisher Scientific |
| AMPure XT beads | Beckman Coulter Genomics |
| flow cytometry staining buffer | Thermo Fisher Scientific |
| **Software** |  |
| CD-HIT software | V4.5.8 |
| DIAMOND software | V0.9.9 |
| SOAPdenovo software | V2.04 |
| MetaGeneMark | V2.10 |
| ANY-maze Video Tracking Software | Stoelting Co. |
| SPSS | Version 20.0, SPSS Inc, IBM |
| Graphpad | Prism9, GraphPad Software |
| R | V4.0.2 |
| PICRUSt2 software | V2.2.0b |
| Image J | NIH, MD |
| Flowjo | V10, Treestar |
| SILVA database | Release 138 |
| DADA2 | qiime2 (2019.7) |
| **Instruments** |  |
| Bioelectrical impedance analysis (BIA) | InBody 120, Seoul, Korea |
| Dual-energy X-ray absorptiometry (DXA) | Horizon, Hologic, Marlborough, MA, USA |
| grip strength dynamometer | 5030JI, JAMAR, Bolingbrook, IL, USA |
| Agilent2100 Bioanalyzer | Agilent, USA |
| Illumina NovaSeq 6000 platform | Illumina, Inc., CA, USA |
| anaerobic chamber | Bugbox Plus UM-017, Baker, USA |
| LeicaDM1000 LED microscope | Leica, Germany |
| Qubit | Invitrogen, USA |
| 7000D mass spectrometer | Agilent, USA |
| LC-MS system | 1290 Infinity LC UPLC coupled to 6545 Q-TOF MS, Agilent, USA |
| GC column DB-FFAP | 30m, 0.25mm, 0.25µm, J&W Scientific, USA |
| UHPLC system | Vanquish, Thermo Fisher Scientific, USA |
| Orbitrap Exploris 120 mass spectrometer | Orbitrap MS, Thermo Fisher Scientific, USA |
| Mouse force gauge | Mark-10 Corporation, USA |
| Ex-vivo muscle function equipment | 800C, Aurora Scientific Inc., Newmarket, Canada |
| Dynamic Muscle Control system | DMC v6.0; Aurora Scientific, Inc., Canada |
| Dynamic Muscle Analysis system | DMA v5.501; Aurora Scientific, Inc., Canada |
| Catwalk | CatWalk XT 9.0, Noldus Information Technology, Netherlands |
| Treadmill | 76-0895, Panlab, Harvard Apparatus, MA, USA |
| Cryostar NX70 microtome | Thermo Fisher Scientific, Waltham, USA |
| fluorescence light microscope system | Leica Microsystems Ltd., Germany |
| animal DXA | XPERT 40 KUBTEC DXA, USA |
| Leica EM UC7 Ultramicrotome | Leica Microsystems Ltd., Germany |
| Transmission electron microscope | Hitachi HT7700, Japan |
| BD FACSAria Fusion Flow Cytometry Cell Sorter | BD Biosciences, USA |
| NanoDrop 2000 | Thermo Fisher Scientific, USA |
| VeritiPro 96-well thermal cycle | applied biosystem, Thermo Fisher Scientific, USA |
| Technology QuantStudio 12K Flex qPCR System | Thermo Fisher Scientific, Waltham, USA |
| **Antibodies** |  |
| anti-MHC I | BA-F8, Developmental Studies Hybridoma Bank |
| anti-MHC IIa | SC-71, Developmental Studies Hybridoma Bank |
| anti-MHC IIb | BF-F3, Developmental Studies Hybridoma Bank |
| goat anti-mouse Alexa Fluor® 350 IgG2b | A21140, Thermo Fisher Scientific |
| goat anti-rabbit Alexa Fluor® 488 IgG1 | A21121, Thermo Fisher Scientific |
| goat anti-rabbit Alexa Fluor® 555 IgM | A21426, Thermo Fisher Scientific |
| anti-Laminin 2 alpha antibody | ab11576, abcam |
| goat anti-rat IgG H&L preabsorbed | ab7094, abcam |
| anti-Muc2 | ab272692, abcam |
| anti-Claudin-1 | ab15098, abcam |
| anti-Occludin | A2601, abclonal |
| anti-E-cadherin | 3195T, Cell Signaling Technology |
| anti-OPA1 | A9833, Abclonal |
| anti-DRP1 | A21968, Abclonal |
| anti-MFN1 | A21293, Abclonal |
| anti-MFN2 | A19678, Abclonal |
| anti-CPT1A | 15184-1-AP, Proteintech |
| anti-ATP5A1 | A11217, Abclonal |
| anti-Cytochrome c oxidase | A11631, Abclonal |
| anti-NRF1 | A5547, Abclonal |
| anti-PGC1α | A12348, Abclonal |
| anti-Histone H3 | A2348, Abclonal |
| anti-FoxO3a | 2497, Cell signaling Technology |
| anti-phospho-FoxO3a | 9466S, Cell Signaling |
| anti-Fbxo32 | A3699, Abclonal |
| anti-Trim63 | A3101, Abclonal |
| anti-β-actin | AC026, Abclonal |
| anti-GAPDH | MA5-15738, Invitrogen |
| anti-rabbit IgG HRP-linked antibody | Cell signaling Technology |
| goat anti-mouse IgG (H + L) antibody | Invitrogen |
| Fc receptor binding inhibitor antibodies | eBioscience™ |
| anti-CD3, PerCP/Cyanine5.5 | 100217, BioLegend |
| anti-CD4, FITC | 100405, BioLegend |
| anti-CD8, APC/Fire 750 | 100765, BioLegend |
| anti-IFNγ, Alexa Fluor® 647 | 505816, BioLegend |

**References**

Charlson, M. E., Carrozzino, D., Guidi, J., & Patierno, C. (2022). Charlson Comorbidity Index: A Critical Review of Clinimetric Properties. *Psychother Psychosom, 91*(1), 8-35. doi:10.1159/000521288

Cox, B., & Emili, A. (2006). Tissue subcellular fractionation and protein extraction for use in mass-spectrometry-based proteomics. *Nat Protoc, 1*(4), 1872-1878. doi:10.1038/nprot.2006.273

Klein, A. B., Nicolaisen, T. S., Ortenblad, N., Gejl, K. D., Jensen, R., Fritzen, A. M., . . . Clemmensen, C. (2021). Pharmacological but not physiological GDF15 suppresses feeding and the motivation to exercise. *Nat Commun, 12*(1), 1041. doi:10.1038/s41467-021-21309-x

Liu, C., Cheng, K. Y., Tong, X., Cheung, W. H., Chow, S. K., Law, S. W., & Wong, R. M. Y. (2023). The role of obesity in sarcopenia and the optimal body composition to prevent against sarcopenia and obesity. *Front Endocrinol (Lausanne), 14*, 1077255. doi:10.3389/fendo.2023.1077255

Liu, C., Wong, P. Y., Tong, X., Chow, S. K., Hung, V. W., Cheung, W. H., . . . Wong, R. M. Y. (2022). Muscle plays a more superior role than fat in bone homeostasis: A cross-sectional study of old Asian people. *Front Endocrinol (Lausanne), 13*, 990442. doi:10.3389/fendo.2022.990442

Sui, H., Wang, S., Liu, G., Meng, F., Cao, Z., & Zhang, Y. (2022). Effects of Heat Stress on Motion Characteristics and Metabolomic Profiles of Boar Spermatozoa. *Genes (Basel), 13*(9). doi:10.3390/genes13091647

Vandeputte, D., Falony, G., Vieira-Silva, S., Tito, R. Y., Joossens, M., & Raes, J. (2016). Stool consistency is strongly associated with gut microbiota richness and composition, enterotypes and bacterial growth rates. *Gut, 65*(1), 57-62. doi:10.1136/gutjnl-2015-309618

Vaughan, K., & Miller, W. C. (2013). Validity and reliability of the Chinese translation of the Physical Activity Scale for the Elderly (PASE). *Disabil Rehabil, 35*(3), 191-197. doi:10.3109/09638288.2012.690498

Wang, J., Cui, C., Chim, Y. N., Yao, H., Shi, L., Xu, J., . . . Cheung, W. H. (2020). Vibration and beta-hydroxy-beta-methylbutyrate treatment suppresses intramuscular fat infiltration and adipogenic differentiation in sarcopenic mice. *J Cachexia Sarcopenia Muscle, 11*(2), 564-577. doi:10.1002/jcsm.12535

Wong, S. H., Zhao, L., Zhang, X., Nakatsu, G., Han, J., Xu, W., . . . Yu, J. (2017). Gavage of Fecal Samples From Patients With Colorectal Cancer Promotes Intestinal Carcinogenesis in Germ-Free and Conventional Mice. *Gastroenterology, 153*(6), 1621-1633 e1626. doi:10.1053/j.gastro.2017.08.022
